# Genomic and Epidemiological Investigations Reveal Chromosomal Integration of the Acipenserid Herpesvirus 3 Genome in Lake Sturgeon *Acipenser fulvescens*

**DOI:** 10.3390/v17040534

**Published:** 2025-04-05

**Authors:** Sharon Clouthier, Umberto Rosani, Arfa Khan, Qiuwen Ding, Eveline Emmenegger, Zhuozhi Wang, Thomas Nalpathamkalam, Bhooma Thiruvahindrapuram

**Affiliations:** 1 Department of Fisheries and Oceans, Freshwater Institute, Winnipeg, MB R3T 2N6, Canada; arfa.khan@dfo-mpo.gc.ca (A.K.); bryanqiuwen.ding@dfo-mpo.gc.ca (Q.D.); 2 Department of Biology, University of Padova, 35131 Padua, Italy; umberto.rosani@unipd.it; 3 U.S. Geological Survey, Western Fisheries Research Center, Seattle, WA 98115, USA; eemmenegger@usgs.gov; 4 The Centre for Applied Genomics, The Hospital for Sick Children, Toronto, ON M5G 1H3, Canada; zwang@sickkids.ca (Z.W.); thomas.nalpathamkalam@sickkids.ca (T.N.); bthiruv@sickkids.ca (B.T.)

**Keywords:** Lake Sturgeon, *Alloherpesviridae*, endogenous, germline, herpesvirus

## Abstract

DNA sequence from a new alloherpesvirus named acipenserid herpesvirus 3 (AciHV-3) was found in sturgeon species that are vulnerable to decline globally. A study was undertaken to develop a better understanding of the virus genome and to develop diagnostic tools to support an epidemiological investigation. A 184,426 bp genome was assembled from PacBio HiFi sequences generated with DNA from a Lake Sturgeon *Acipenser fulvescens* gonad cell line. The AciHV-3 genome was contiguous with host chromosomal DNA and was structured with telomere-like terminal direct repeat regions, five internal direct repeat regions and a U region that included intact open reading frames encoding alloherpesvirus core proteins. Diagnostic testing conducted with a newly developed and analytically validated qPCR assay established the ubiquitous presence and high titer of AciHV-3 DNA in somatic and germline tissues from wild Lake Sturgeon in the Hudson Bay drainage basin. Phylogenetic reconstructions confirm that the monophyletic AciHV-3 lineage shares a common ancestor with AciHV-1 and that AciHV-3 taxa cluster according to their sturgeon host. The same genotype of AciHV-3 is found in disjunctive Lake Sturgeon populations within and among drainage basins. The results support the hypotheses that AciHV-3 has established latency through germline chromosomal integration, is vertically transmitted via a Mendelian pattern of inheritance, is evolving in a manner consistent with a replication competent virus and has co-evolved with its host reaching genetic fixation in Lake Sturgeon populations in central Canada.

## 1. Introduction

Herpesviruses (HVs) are large double-stranded DNA viruses that have been categorized into three families—*Ortho-*, *Allo-* and *Malacoherpesviridae*—on the basis of their genome sequences [1]. Membership in the first family is restricted to HVs of mammals, birds and reptiles whereas fish and amphibian HVs have been assigned to the second family, and the third family is comprised of HVs of mollusks, such as bivalves and gastropods [1,2,3]. Herpesvirus genomes range in size from 124 to 295 kilobase pairs (kbp) and contain various combinations of unique (U) regions and repeat elements. A total of six major classes of genome structure have been identified [4]. Despite this genetic disparity, almost all herpesviruses have a biphasic lifecycle, alternating between a latent state and an actively replicating phase. Active replication of herpesvirus DNA occurs through a rolling circle mechanism in which concatemers of the virus genome are synthesized and then cut into single genome units prior to being packaged into the virus capsid [5]. Latency is typically established extrachromosomally via the formation of silent circular episomes of herpesvirus DNA in the nucleus of specific somatic cells [6]. In the case of a select few herpesviruses [7], latency occurs via chromosomal integration of the virus genome in somatic (Epstein-Barr virus (EBV) [8]; Marek’s disease virus (MDV) [9]) or germ cells (human herpesvirus 6 (HHV-6) [10]). Integration in germinal cells results in vertical transmission of the virus through inheritance of the parental genome, hence the name inherited chromosomally integrated HHV-6 (iciHHV-6). Reactivation of latent herpesviruses results in exogenous replicating virions as shown for MDV [11], EBV [12,13] and HHV-6 [10]. Infections with iciHHV-6 are uniquely characterized by their apparent high virus loads (i.e., DNAemia), ubiquitous presence of virus DNA in all tissues and a Mendelian pattern of inheritance [14].

Herpesviruses that are phylogenetically congruent with the *Alloherpesviridae* family pose a disease threat to extant sturgeon species. Sturgeon (*Acipenseridae*) and their sister lineage paddlefish (*Polyodontidae*) are considered ancient fish that descend from a common ancestor and are taxonomically grouped within the order Acipenseriformes [15]. Concern has grown for this taxonomic group as the majority of species have been listed as Critically Endangered by the International Union for Conservation of Nature Red List of Threatened Species [16]. These fish exhibit complex genomes with macro- and micro-chromosomes [17], a slow rate of molecular evolution as evidenced by their primitive features [18] and complex polyploid genomes with chromosome numbers ranging from approximately 120 to 520 [17,19,20,21]. Lake Sturgeon *Acipenser fulvescens* with 240–268 chromosomes is one of the species found in North America and is distributed in rivers and lakes across its range [22,23]. For the purposes of sustainable conservation and management, populations in Canada have been divided into four designatable units (DUs) based on their phylogeographic genetic structure: DU1—Western Hudson Bay; DU2—Saskatchewan–Nelson River; DU3—Southern Hudson Bay–James Bay; and DU4—Great Lakes–Upper St. Lawrence [24]. The impact of herpesvirus disease on these biodiverse Lake Sturgeon populations remains unknown.

Three lineages of sturgeon HVs have been identified—acipenser(id) herpesvirus 1 to 3 (AciHV-1 to 3)—and are grouped into two genera—*Ictavirus* (AciHV-2) [1] or the proposed genus *Acivirus* (AciHV-1 and AciHV-3) [25]. The AciHV-2 lineage appears to have evolved as a result of a host jump from an Ictalurid species, but extant disease events have only been reported in sturgeon [26,27,28,29,30,31]. AciHV-1 was originally isolated from the California White Sturgeon *A. transmontanus* [32] and has been proposed to represent an unclassified lineage within the *Alloherpesviridae* [33,34]. Herpesviruses closely related to AciHV-1 have been described and/or isolated from Lake Sturgeon including lake sturgeon herpesvirus (LSHV) [35] and lake sturgeon herpesvirus 2 (LSHV-2) [36]. These two viruses cluster with AciHV-1 in a monophyletic clade [25,36] that appears to reflect host–virus coevolution within different sturgeon species and, contrary to other groups [35,36], is parsimoniously interpreted herein to be isomorphic with the virus species taxonomic level corresponding to AciHV-1 [25]. Koch’s postulates have been fulfilled for AciHV-1 [32], LSHV-2 [36] and AciHV-2 [26] but not AciHV-3 [25]. Using molecular techniques, AciHV-3 DNA has been detected in six sturgeon species: *A. fulvescens, A. transmontanus,* Sterlet Sturgeon *A. ruthenus,* Pallid Sturgeon *Scaphirhynchus albus,* Shortnose Sturgeon *A. brevirostrum* and Atlantic Sturgeon *A. oxyrinchus oxyrinchus* [25]. Sturgeon herpesviruses, including those from Lake Sturgeon [35,36], are associated with skin infections in which the disease manifests as focal hyperplasia that can progress to acute systemic infections [37]. Host age, virus dose and environmental conditions such as stocking density and water temperature are among the risk factors that influence the incidence and severity of disease [26,31,36,38]. In the case of mild infections, lesions can appear and then regress with no apparent negative effect on the sturgeon [25]. The reappearance of lesions in convalescent sturgeon suggests that these viruses can establish persistent infections, likely by establishing latency in a manner similar to other herpesviruses [26]. Spread of the viruses through populations can occur by horizontal or possibly vertical transmission pathways [26,31]. Diagnosis of alloherpesvirus infections has occurred through microscopy [26,31,32,35,36,37,39], virus isolation [36,39,40], molecular assays [25,29,33,34,35,36,41,42] and next generation sequencing (NGS) methods [25,35,36,41]. Since AciHV-3 DNA has been identified in naïve sturgeon cell lines [25], virus isolation performed with extant sturgeon cell lines should be used with caution or avoided.

Our investigations of regional Lake Sturgeon populations in Canada led us to the discovery of AciHV-3 DNA in juvenile Lake Sturgeon displaying epithelial skin lesions [25]. The fish were progeny of wild broodstock and were being reared in a hatchery prior to release into their natal river for conservation stocking purposes. Histopathology of the lesions revealed herpesvirus-like cellular changes including epithelial hyperplasia. Given that the cell culture results were inconclusive and difficult to interpret, the diagnosis of AciHV-3 as an alloherpesvirus was made through NGS results and phylogenetic analyses based on concatenations of five core alloHV proteins [25]. In our second study reported herein, we extend our initial findings using a long read NGS approach to sequence the whole genome of AciHV-3 from a primary Lake Sturgeon gonad cell line (LSGO) [43], validate a newly developed diagnostic quantitative PCR (qPCR) assay and use the test to investigate the biology and epidemiology of AciHV-3. The results reveal the unique lifecycle features of an endogenous virus integrated into the chromosome of germline cells (i.e., referred to herein as a latent infection) and suggest that ciAciHV-3 may be capable of establishing an exogenous phase given its apparent evolution under purifying selection. The high prevalence of AciHV-3 DNA in wild Lake Sturgeon populations that share a common genotype of the virus despite being geographically separated suggest a long-term host–virus relationship in which the virus genome has reached fixation as a genetic trait.

## 2. Materials and Methods

### 2.1. Field Study of Lake Sturgeon in the Hudson Bay Drainage Basin

**Figure 1:**
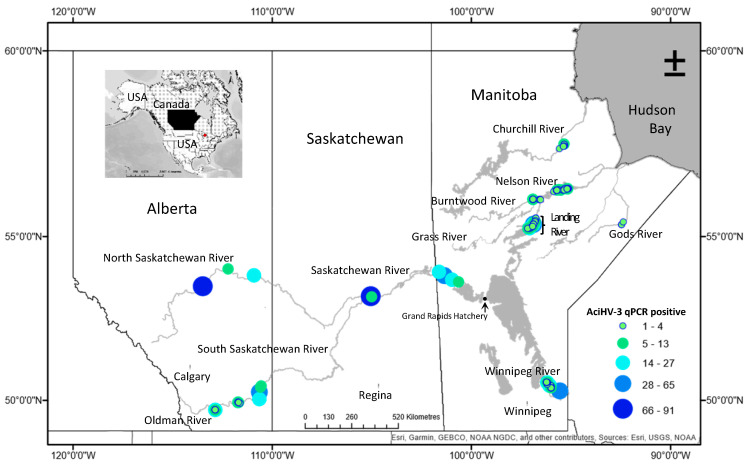
Study area map (2010–2021) showing geographic distribution of acipenserid herpesvirus 3 (AciHV-3) qPCR-positive Lake Sturgeon *Acipenser fulvescens* in the Hudson Bay drainage basin (HBDB). A map inset of North America provides the relative location of the HBDB study area (black) in Canada (stipple) with the red dot marking the location where samples were collected in 2019 from Lake Sturgeon in Lake Superior. The map was created using ESRI [44] ArcGIS 10.8.1 software with publicly available CANVec Series shapefiles from Natural Resources Canada [45]. AciHV-3 DNA prevalence levels and number of fish tested annually from each river across the HBDB are available in Table S1.

The field study area encompassed ten rivers of the Hudson Bay drainage basin in central Canada (Figure 1). Lake Sturgeon populations in the basin have been designated Threatened (DU1 Western Hudson Bay, DU2 Saskatchewan–Nelson River) or Special Concern (DU3 Southern Hudson Bay–James Bay) by the Committee on the Status of endangered Wildlife in Canada [24].

Non-lethal sampling of pectoral fin tissue (0.5-cm^2^) from wild Lake Sturgeon (n = 1162) in the basin occurred between 2010 and 2021 (Figure 1). Fin tissues were removed using aseptic technique and preserved using RNAlater^®^ (Life Technologies, Carlsbad, CA, USA). Specimens were held at ambient temperature for 24 h, frozen at −20 °C and then shipped on ice to the Freshwater Institute (Winnipeg, Manitoba) where they were stored at −80 °C. Gametes (milt, n = 17 males; eggs, n = 21 females) were collected from sturgeon spawning in the Winnipeg, Landing or Burntwood Rivers between 2011 and 2014. Samples were flash frozen in liquid nitrogen or a dry ice–ethanol bath, transported on dry ice and then stored at the Freshwater Institute in ultracold freezers. Winnipeg River offspring (n = 60) reared at the University of Manitoba Aquatic Animal Health Facility (Winnipeg, Manitoba, Canada) were euthanized by an overdose of sodium bicarbonate (0.1%)-buffered anesthetic tricaine methanesulfonate (MS-222) at 46, 48 or 74 days post-fertilization and immediately frozen at −80 °C. Juvenile Lake Sturgeon from the 2017 (n = 10) and 2019 (n = 3) year classes of wild progeny reared at the Grand Rapids Hatchery (Grand Rapids, Manitoba, Canada) were similarly euthanized and then transported to the Freshwater Institute where they were stored at −80 °C prior to removal of their tissues for the virus tropism study.

Sample collection was coordinated with groups conducting Lake Sturgeon monitoring studies as described previously in Clouthier et al. [46]. Additional samples were collected from Lake Sturgeon in the Oldman and South Saskatchewan Rivers in 2021 (May and June) under the direction of H. Kristine Wilson, John Derksen, Nick Savidov (Lethbridge College, Lethbridge, AB, Canada) and Shane Petry (Alberta Environment and Parks, Lethbridge, AB, Canada). In this case, sturgeon were caught using catch-and-release angling, and catch sites were identified with Global Positioning System (GIS) coordinates. Larval and juvenile offspring of wild Lake Sturgeon broodstock were reared at the University of Manitoba Aquatic Animal Holding Facility under the guidance of Gary Anderson (University of Manitoba) and at the Grand Rapids Hatchery under the purview of Cheryl Klassen (Manitoba Hydro, Winnipeg, MB, Canada).

Sturgeon populations were not sampled every year, and the number of samples collected from each population ranged from seven in each of Gods and Grass Rivers to one hundred and thirty-two in the Saskatchewan River (Table S1).

### 2.2. Other Sample Collections

Tissue samples were also collected from other sturgeon and fish species and preserved in RNAlater^®^ (Life Technologies) for molecular testing. Unless otherwise specified, all samples from each collection were analyzed using the Q1mcp test. Pectoral fin tissue samples (n = 63) were collected from wild White Sturgeon in the Upper Columbia River in British Columbia (Canada). Fish were caught using catch and release angling in 2017 (September and October) as part of a conservation program and under the direction of Chad Fuller (Okanagan Nation Alliance, Westbank, BC, Canada). One of these samples was analyzed. Wild Lake Sturgeon from either Goulais Bay (n = 87) or Batchawana Bay (n = 4) of Lake Superior in Ontario (Canada) were captured using gill nets in 2019 (July and August) and non-lethal pectoral fin tissue samples were removed under the direction of William Gardner (Fisheries and Oceans Canada, Sault Ste Marie, ON, Canada). A total of ten samples were analyzed. Wild adult Spotted Gar *Lepisosteus oculatus* (n = 7) and Longnose Gar *L. osseus* (n = 3) from Clear Lake, Black Lake or Cane River Lake in Louisiana (USA) were caught in 2022 (February or April) using either gill nets or electrofishing methods by Villis Dowden, Brittainey Thaxton and Tory Robicheaux of the Louisiana Department of Wildlife and Fisheries (Natchitoches, LA, USA). Non-lethal pectoral fin tissue samples were collected by Emmet Guy, Lindsey Adams, Brett Hortman, Greg Landry, Wade Scarbrough and Steve Carbin of the U.S. Fish and Wildlife Service (Natchitoches, LA, USA). In 2022, cultured White Sturgeon (n = 76) held at the International Centre for Sturgeon Studies were euthanized and sampled under the direction of Barry Milligan as part of a broader project (Vancouver Island University (Nanaimo, BC, Canada)). Pectoral fin tissue from one individual was analyzed. Juvenile progeny (n = 10) of wild American Paddlefish *Polyodon spathula* broodstock were reared by Nick Starzl at the Gavins Point National Fish Hatchery (Yankton, SD, USA) in 2023, euthanized and sent on ice to Montana where pectoral fin (n = 9) and kidney tissues (n = 10) were removed using aseptic technique by Lacey Hopper (U.S. Fish and Wildlife Service (Bozeman, MT, USA)). Non-invasive mucus samples from captive Green Sturgeon *A. medirostris* (n = 5) held at the Putah Creek Facility at the University of California Davis Center for Aquatic Biology and Aquaculture (Davis, CA, USA) were collected in 2024 by researchers Nann Fangue, Sarah Baird, Emily Funk and Andrea Schreier (University of California Davis (CA, USA)).

### 2.3. Viruses and General Virology

The viruses included in this study are presented in Table S2. The rhabdoviruses were cultured on *epithelioma papulosum cyprini* (EPC) cells at 15 °C (infectious hematopoietic necrosis virus (IHNV) 93–057 and viral hemorrhagic septicemia virus (VHSV) 99–292) or 20 °C (spring viremia of carp virus (SVCV) HHOcarp06) [47,48]. The birnavirus infectious pancreatic necrosis virus (IPNV) Jasper was cultured at 15 °C on Chinook Salmon embryo cells (CHSE-214) [49] and the alloherpesviruses were amplified at the same temperature on Common Carp brain (cyprinid herpesvirus 3 (CyHV-3) F98-50) or White Sturgeon spleen (WSS-2) (shortnose (SN) AciHV-2) cell lines [32,50]. Naïve (i.e., non-inoculated) White Sturgeon skin (WSSK [32]), WSS-2 [40] and LSGO [43] cell lines were the source of AciHV-3 WSSK, AciHV-3 WSS2 or AciHV-3 LSGO, respectively. The cell lines were amplified using Minimal Essential Medium supplemented with 2 mM L-glutamine, Hank’s salts and 2 to 10% fetal bovine serum (Gibco, Thermo Fisher Scientific, Waltham, MA, USA). Where applicable, Antibiotic-Antimycotic (Gibco, Thermo Fisher Scientific) was added following virus adsorption. Cell monolayers infected with IHNV, VHSV, SVCV, IPNV, CyHV-3 or SN AciHV-2 were inoculated using a multiplicity of infection between 0.01 and 0.0001, and the virus was harvested from monolayers displaying complete cytopathic effects. The whole cell lysates were stored at −80 °C.

The term “isolate” will be restricted to those viruses that have been amplified in cell culture. Virus taxa that have nucleotide sequence information but have not been isolated by cell culture will be referred to by their given name, and instead of “isolate”, terms such as “DNA”, “sequence” or “genome” will be applied (e.g., AciHV-3 DNA). Taxa such as AciHV-3 LSGO with a genome that has been shown to be chromosomally integrated may also be referred to as ciAciHV-3 LSGO.

### 2.4. Viral Nucleic Acid

#### 2.4.1. DNA Synthesis and Plasmid Purification

Partial DNA sequences encompassing nucleotides 1200 to 1800 of the major capsid protein (mcp) DNA sequence from AciHV-1 UC Davis, LSHV and LSHV-2 were synthesized as 600 bp GeneArt Strings™ DNA fragments by Life Technologies l (Table S2). Full-length major capsid protein DNA sequences for representative alloherpesviruses and herpesviruses were synthesized and inserted into vector pJ248 (ATUM) (Table S2). Similarly, synthetic DNA encoding the artificial positive control (APC) sequence for pLSAciHV3-APC-Q1mcp was placed in vector pJ204 (ATUM) (2794 bp, 3.3 × 10^11^ copies per μg DNA; Figure S1). The construct was designed to include the Q1mcp primer and probe sequences as well as a sequence complementary to the APC-M probe as outlined by Snow et al. [51]. Plasmid DNA was transformed into MAX Efficiency^®^ *Escherichia coli* DH5α Competent Cells according to the manufacturer’s instructions (Invitrogen, Carlsbad, CA, USA) and purified from *E. coli* overnight cultures using the QIAprep Spin Miniprep Kit as described by Qiagen (Venlo, The Netherlands).

#### 2.4.2. Nucleic Acid Extraction

Infected cell culture lysates of CyHV-3 F98-50, IHNV 93-057, IPNV Jasper, SVCV HHOcarp06 and VHSV 99-292 were used to prepare nucleic acid for the analytical validation study. DNA was purified from CyHV-3 lysates, and RNA was extracted and reverse transcribed using the methods described in Clouthier et al. [52,53]. Genomic DNA for the validation study was extracted from naïve CHSE, EPC, WSSK and WSS-2 cell lines using either DNAzol^TM^ Reagent (Thermo Fisher Scientific) or the DNeasy Blood & Tissue Kit (Qiagen) following the manufacturers’ instructions.

Naïve Lake Sturgeon gonad cell monolayers (n = 2 × 175 cm^2^) were processed to prepare high molecular weight (HMW) DNA for high fidelity (HiFi) PacBio long read sequencing and the validation study using the Monarch^®^ HMW DNA Extraction Kit for Cells (New England BioLabs, Ipswich, MA, USA) as described by the manufacturer. DNA quality and quantity were assessed using the Qubit 4.0 fluorometer (Invitrogen, Burlington, ON, Canada) and NanoDrop 8000 spectrophotometer (Thermo Fisher Scientific, Mississauga, ON, Canada).

Tissue samples collected in the field from wild Lake Sturgeon (fin, eggs and milt) or gar (fin), from captive Green Sturgeon (mucus) or White Sturgeon (fin) broodstock, from hatchery-reared offspring of wild sturgeon (pectoral fin, brain, mouth, barbels, gills, heart, esophagus, kidney, spleen, intestine, liver, muscle, skin and whole larvae) and from American Paddlefish (fin) were used to prepare DNA for analysis with the Q1mcp assay. Samples were processed as described in Clouthier et al. [25] except that in some cases, the DNA was extracted on the KingFisher Flex Purification System (Thermo Fisher) using the MagMax™ Pathogen RNA/DNA Kit (Applied Biosystems™, Foster City, CA, USA) or manually using the Zymo Quick-DNA/RNA Microprep Plus Kit (Zymo Research, Irvine, CA, USA). Each qPCR test was run with a maximum of 1500 ng DNA.

### 2.5. Next-Generation Sequencing, Assembly and Annotation

High molecular weight DNA extracted from the naïve LSGO cell monolayers was sent to The Center for Applied Genomics at The Hospital for Sick Children (Toronto, ON, Canada) where library preparation, next-generation sequencing and read assembly were performed.

A single library was constructed using the PacBio^®^ SMRTbell^®^ (Single Molecule, Real-Time Sequencing) Express Template Prep Kit 2.0 (Pacific Biosciences, Menlo Park, CA, USA) using 6.8 μg of HMW LSGO DNA as the starting template. Size selection of the library to remove molecules shorter than 5 kbp was performed with the Ampure^®^ PB beads (Pacific Biosciences). Library quality was assessed with Agilent Genomic DNA ScreenTape run on the 4200 TapeStation system (Agilent Technologies, Santa Clara, CA, USA), and quantity was determined on the Qubit 4.0 fluorometer (Invitrogen) using the 1× dsDNA High-Sensitivity Assay Kit (Invitrogen). The library was loaded onto two SMRT Cells at a concentration of 100 pM. Sequencing was performed on the PacBio Sequel^®^ IIe sequencing platform using the Sequel^®^ II sequencing kit 2.0 (Pacific Biosciences). Long high-fidelity reads were generated in the circular consensus sequencing mode with 16 full-pass subreads and 30 h movie times. Barcode adaptors were removed from the polymerase reads with the demultiplexing algorithm LIMA from Pacific Biosciences. The sequencing metrics per SMRT cell revealed a yield of 3,046,622 and 2,873,793 raw HiFi reads totaling 26,787,454,926 and 24,844,285,587 bp with quality scores > Q20 (i.e., median Q38 and Q39) and mean read lengths of 8792 and 8645 bp. Sample barcode and adapter sequences were removed from the raw HiFi reads with the demultiplexing algorithm Lima v2.6.0 from Pacific Biosciences. The number of trimmed reads remaining after the removal of bar codes was 2,739,166 and 2,582,224.

De novo assembly of the pooled HiFi reads using the hifiasm (v0.16.1) assembler resulted in 16,219 contigs (length > 10 kbp) of which 649 were >1 Mbp in length. The assembly span was 3,515,768,025 bp, mean contig length was 216,768 bp and the assembly N50 was 614,989 bp. Only those contigs determined by BLASTN analysis to be positive for the AciHV-3 sequence reported in Clouthier et al. [25] were analyzed further.

The genome was searched for sequences similar to the cis-acting elements pac1 and pac2 of HHV-6 using the following search criteria. Putative pac1 sequences were identified by searching for two clusters of 5–7 consecutive C nucleotides separated by 4 to 20 bp of A- or T-rich sequence (i.e., ≥60% A and/or T). Putative pac2 sequences were identified using two methods. The first approach searched for the sequence “CGCGGCG” and looked for 5–10 consecutive A nucleotides within 100 bp of the match. The second, less stringent approach, searched for five consecutive A nucleotides and then looked for the sequence “CGCGGCG” within 100 bp allowing for at most one mismatch.

Open reading frames (ORFs) initiated with ATG, ending with TAA, TGA or TAG and longer than 150 bp were identified using Geneious Prime v2023.2.1 (Dotmatics, Boston, MA, USA), SeqBuilder Pro^TM^ module of Lasergene v17.5.0 (DNASTART, Inc, Madison, WI, USA), GenemarkS [54] and Genome Annotation Transfer Utility [55] software. Sequences were manually annotated and visualized in Geneious Prime v2023.2.1 (Dotmatics, Boston, MA, USA). DNA sequences were analyzed for repeats using RepeatMasker v4.0.9 [56] and the Dfam v3.8 database [57] and the Repeat Finder plugin in Geneious Prime v2023.2.1 (Dotmatics). AciHV-3 protein sequences ≥50 amino acids were screened against the National Center for Biotechnology Information (NCBI) non-redundant (nr) or the ClusteredNR databases (all taxa or restricted to Herpesvirales or *Retroviridae* taxa) using the protein–protein BLAST (BLASTP) and position-specific iterated BLAST (PSI-BLAST) (expected value ≤ 0.01) algorithms v2.13.0 to 2.16.0 to identify potential homologues [58,59]. Putative donor and acceptor splice sites were predicted with Spliceator v2.1 (model 600, ≥97%) [60], DeepTMHMM v1.0.14 to 1.0.42 [61] was used to identify signal peptides and transmembrane domains and the SUPERFAMILY database [62,63] was screened for structural protein domains with amino acid sequences translated from predicted AciHV-3 ORFs. OrthoFinder v2.5.4 [64] was used to identify orthologous genes from the proteomes of AciHV-3 LSGO and 128 herpesvirus species retrieved from NCBI (Data S1). This software defaults to FastTree for inferring maximum likelihood phylogenetic trees from MAFFT multiple sequence alignments. Distant evolutionary relationships of AciHV-3 proteins displaying no homology to proteins in NCBI were identified using HHblits [65]. Conserved features of these proteins were also found using the NCBI Conserved Domain Database v3.20, 3.21 (CDD) [66,67]. The ORFs were named using the prefix AFA (*Acipenser fulvescens* contig A) followed by a number starting with 1. An exception to this naming system was used for ORFs detected in internal repeat region (IRR) 2, in which case, the prefix R2 was followed by a number starting with 1.

### 2.6. Genome Nucleotide Sequence Accession Number

The AciHV-3 LSGO genome sequence has been deposited in the NCBI Genbank database under accession number PQ564448. The representative genome sequence originated from contig ptg001728l and included the terminal repeats and complete U region.

### 2.7. Evaluation of Alloherpesvirus Core Proteins

Three proteins that are critical for virion morphogenesis (capsid triplex protein 2, capsid maturation protease and DNA packaging terminase subunit 1) were selected to investigate whether the virus genome encoded intact genes compatible with virion formation. Full-length amino acid sequences of each protein were retrieved from the NCBI Genbank protein database for LSHV, ranid HV 3 (RaHV-3), CyHV-3, ictalurid HV 1 (IcHV-1), Teratorn 7319, HHV-1, and HHV-4 AG876 (Table S2). The sequences were aligned in Geneious Prime v2023.2.1 using the algorithm MAFFT v7.490 [68,69]. Secondary structure prediction of the proteins was performed using the EMBOSS 6.5.7 tool garnier, which implements the Garnier Osguthorpe Robson algorithm (GOR I) in Geneious Prime v2023.2.1. Conserved amino acids of structural or functional significance were identified from the literature for the capsid triplex protein 2 [70,71], capsid maturation protein [72] and the terminase [73,74,75].

DNA sequences encoding the twelve core alloherpesvirus proteins from the AciHV-3 LSGO genome were compared to sequences encoding homologues from two contigs containing the full virus genome sequence—ptg001881l and ptg009622l. The sequences were evaluated for polymorphisms to see if the open reading frames were intact. The DNA sequences were aligned in Geneious Prime v2023.2.1 using MAFFT v7.490 [68,69]. After annotating the AciHV-3 LSGO ORF and selecting it as the reference sequence, variations in the other two sequences were identified in Geneious Prime v2023.2.1 for each of the twelve core ORFs.

### 2.8. PCR Tests

#### 2.8.1. qPCR Test (Q1mcp)

A qPCR test targeting 67 bp (nucleotides 1453–1502) of the gene encoding the major capsid protein of Lake Sturgeon AciHV-3 was developed to screen samples for detection of AciHV-3 DNA. The nucleotide sequence for the forward primer LSBVmcpqF2 was 5′ GTCGCCGAAATCACCTTGA 3′, and for the reverse primer LSBVmcpqR2, it was 5′ TGGTGGCGTCGAACATCTC 3′ (MilliporeSigma, Burlington, MA, USA). The probe for the Q1mcp assay was LSBVmcpqP2 (5′ FAM AGGTACGGCAACCAC MGBNFQ 3′), and the positive control probe was APC-M (5′ VIC ACCGTCTAGCATCCAGT MGBNFQ 3′) (Life Technologies). Each 25 μL reaction included 0.8 μM LSBVmcpqF2, 0.8 μM LSBVmcpqR2, 0.25 μM LSBVmcpqP2, 0.20 μM APC-M probe, 1x Taqman Universal Mastermix (Applied Biosystems) and ≤1500 ng DNA.

Each set of unknown samples was run with positive control samples P1 (15 mg tissue plus 5 × 10^5^ copies of pLSAciHV3-APC-Q1mcp), P3A (5 × 10^4^ copies of pLSAciHV3-APC-Q1mcp) and P3B (5 × 10^2^ copies of pLSAciHV3-APC-Q1mcp) and negative control samples N1 (ATL (Qiagen) buffer) and N3 (water). Including these samples in each run provided a mechanism to check that the expected results were obtained for the nucleic acid extraction (P1 and N1) and qPCR (P3A, P3B and N3) steps.

The Q1mcp tests were run in replicate (n = 3 to 5) per sample in 96-well plate format on the Stratagene Mx3000/5P platform (Agilent Technologies). The cycling conditions were 1 cycle of 50 °C for 2 min, 1 cycle of 95 °C for 10 min and 40 cycles of 95 °C for 15 s and 60 °C for 1 min. The term cycle threshold (Ct) was used here to refer to the PCR cycle at which Q1mcp amplification crosses a threshold and was recorded [76].

The relative quantity of AciHV-3 DNA in each sample was determined using the standard curve method (MxPro v4.10 software) in which the positive control plasmid pLSAciHV3-APC-Q1mcp was 10-fold serially diluted from 5 × 10^7^ to 50 copies. DNAemia (also referred to synonymously as virus or viral DNA load or titer) was expressed as equivalent plasmid copies (epcs) per mg tissue or μg DNA.

#### 2.8.2. Genotyping PCR (GCmcp)

The GCmcp PCR assay was performed as described previously by Clouthier et al., [25]. PCR products of the appropriate molecular weight (536 bp) were purified from 1% agarose gels using the QIAquick Gel Extraction kit (Qiagen) according to manufacturer’s instructions. Sanger sequencing [77] of the purified DNA was conducted by the Sanger Sequencing Facility at the Hospital for Sick Children (Toronto, ON, Canada). The sequence data were analyzed using BioEdit v7.0.5.3 software [78]. Primer sequences were removed resulting in a 493 bp fragment used for genotyping the new AciHV-3 sequences detected in Lake Sturgeon from each waterway and Green Sturgeon from California.

### 2.9. Optimization and Analytical Validation of Q1mcp

#### 2.9.1. qPCR Assay Development and Optimization

The mcp gene was selected as the target molecule for the AciHV-3 qPCR test. At the time the test was being developed, the only known AciHV-3 representative mcp sequence available for primer and probe design was from AciHV-3 LS UNR.

Candidate primers (n = 14) and Taqman™ minor groove binder (MGB) probes (n = 8) were identified within 5 different regions of the AciHV-3 LS UNR major capsid protein gene using AlleleID (v7, PREMIER Biosoft International, San Francisco, CA, USA) and Primer Express (v3.0.1, Applied Biosystems) software. qPCR assays (n = 16) were evaluated with either plasmid, tissue or cell line DNA as the template.

Primer pairs were tested at 900 nM equimolar concentration with SYBR Green (Applied Biosystems) and pLSAciHV-3mcp plasmid DNA as the template. Dissociation curves were generated to assess primer–dimer formation and relative amplicon quantity. Primers were retained if amplification but no primer–dimer was observed.

Candidate qPCR assays were initially run with 900 nM equimolar concentrations of primer and 250 nM probe with pLSAciHV-3mcp plasmid DNA as the template. Tests were ranked based on relative sensitivity and fluorescence such that tests with lower Ct and higher last dRn values were ranked higher. The 5 best assays were selected for further optimization.

These assays were optimized with plasmid and cell line DNA templates using the iterative process of testing primer concentrations from 500 to 900 nM (100 nM increments) while keeping the probe concentration at 250 nM. The top 3 assays displaying the highest sensitivity and fluorescence were then tested using the optimized primer concentration while varying the probe concentration from 100 to 250 nM (50 nM increments). The test with the best performance on both types of template DNA was selected for further analyses in the analytical validation study.

#### 2.9.2. Analytical Validation

The analytical sensitivity of Q1mcp was determined by measuring its 100% limit of detection (LOD) using the standard curve method with serially diluted plasmid DNA (pLSAciHV3-APC-Q1mcp). Assay performance parameters of reaction efficiency and linearity were also evaluated with DNA extracted from AciHV-3-positive sturgeon fin tissue, whole cell lysates of LSGO cell monolayers inoculated with tissue homogenates from sturgeon displaying lesions and another plasmid construct containing the full-length mcp gene from AciHV-3 LSGO (pAciHV-3-mcp). The relative efficiency of Q1mcp with these templates was used to determine if plasmid DNA was a suitable proxy for quantifying AciHV-3 in tissue. Assay efficiency and analytical sensitivity were also assessed to determine if addition of the APC probe (200 or 250 nM) affected Q1mcp performance. Standard curves were generated with DNA 5 or 10-fold serially diluted over 8 to 10 orders of magnitude using 3 replicates per dilution in 1 to 3 runs per template. The 100% LOD corresponded to the lowest concentration of serially diluted DNA for which all replicates had Ct values <40 and was expressed as equivalent plasmid copies. One plasmid copy is assumed to be equivalent to one virus genome copy.

The inclusivity of Q1mcp refers to the ability of the assay to detect AciHV-3 nucleic acid in members of the *Acipenseridae* family. The scope was investigated using DNA isolated from *A. brevirostrum, A. transmontanus*, *A. fulvescens* and *S. albus* fin tissues, *A. medirostris* mucus as well as naïve cell lines from *A. transmontanus* (WSSK, WSS-2; originating from two different laboratories) and *A. fulvescens* (LSGO) (Table S2). The exclusivity or ability of the assay to not detect other alloherpesviruses or potentially co-occurring viruses was evaluated using plasmid DNA (5 × 10^6^ copies) encoding the full length major capsid protein sequence from representatives of the proposed *Acivirus* (LSHV, LSHV-2 and AciHV-1 UC Davis), *Batravirus* (RaHV-1), *Cyvirus* (CyHV-3) and *Ictavirus* (IcHV-1, shortnose sturgeon HV, snake river white sturgeon HV, siberian sturgeon (SbS) HV 1 and SbSHV-2) genera of the *Alloherpesviridae* family (Table S2) and proposed members of the *Mimiviridae* family (namao virus and white sturgeon iridovirus) as well as RNA (30–100 ng) isolated from the cell culture amplified birnavirus IPNV (Jasper) or rhabdoviruses VHSV (99-292), IHNV (93-057) and SVCV (HHOcarp06) (Table S2). The exclusivity study also included DNA samples extracted from Spotted Gar, Longnose Gar and American Paddlefish fin tissue as well as cells derived from fish species outside of the *Acipenseridae* family such as the CHSE and EPC cell lines. The Ct values obtained for each sample (n = 3 replicates) were averaged and then reported as detected (Ct < 40) or not detected (Ct ≥ 40). Estimates of inclusivity and exclusivity were generated with 2-way cross-tabulation tables and reported as a percent agreement between the expected and observed results.

The repeatability of Q1mcp was evaluated using the continuous outcome results obtained from 58 runs of pLSAciHV3-APC-Q1mcp plasmid DNA 10-fold serially diluted from 5 × 10^7^ to 50 copies. The data were displayed as a graph of the intra-run standard deviation of Ct values (3 replicates) plotted against the mean Ct value for each dilution per series. Intra and inter-run repeatability were also assessed using the coefficient of variation (CV) calculated as a ratio of the standard deviation over the average Ct value.

The statistical analyses and graphics described in this section were produced with Stata/SE v17.

### 2.10. Phylogenetic Analyses

Bayesian phylogenies were generated to evaluate the genotypes and evolutionary relationships of AciHV-3 using a subset of the virus sequences presented in Table S2. Virus taxonomy designations were based on the 2023 taxonomy release approved by the International Committee on Taxonomy of Viruses [1].

AciHV-3 taxonomy was investigated using concatenations of five core alloherpesvirus protein sequences—capsid maturation protein, capsid triplex subunit 2, DNA polymerase catalytic subunit, helicase primase helicase subunit and the major capsid protein. Amino acid sequences of each full-length protein were retrieved from the NCBI Genbank protein database and from the new AciHV-3 LSGO genome (Table S2).

AciHV-3 genotypes were determined using a 493 bp DNA fragment of the mcp gene amplified from new sequences in this study or identified from nucleotide sequences in the NCBI Genbank nucleotide databases (Table S2).

Bayesian phylogenetic trees were inferred using the BEAST software package (v1.10.4) [79] run locally or on the CIPRES Science Gateway v3.3 [80] with sequences aligned in ClustalX2 (default parameters) [81]. Suitable nucleotide and amino acid substitution models with the best Bayesian information criterion values [82] were identified through the Molecular Evolutionary Genetics Analysis (MEGA) software (v.11) [83]. Markov-chain Monte-Carlo methods were run for 10 million generations. Diagnostics of >200 estimated sample size [84], convergence and adequate mixing were performed in Tracer v1.7.1 [85] and maximum clade credibility trees were generated in TreeAnnotator (BEAST v1.10.4) [79]. Trees with the lowest posterior mean score were selected for presentation and were drawn in FigTree v1.4.4 [86].

## 3. Results

### 3.1. Identification of Contigs Containing AciHV-3 Sequence

**Figure 2:**
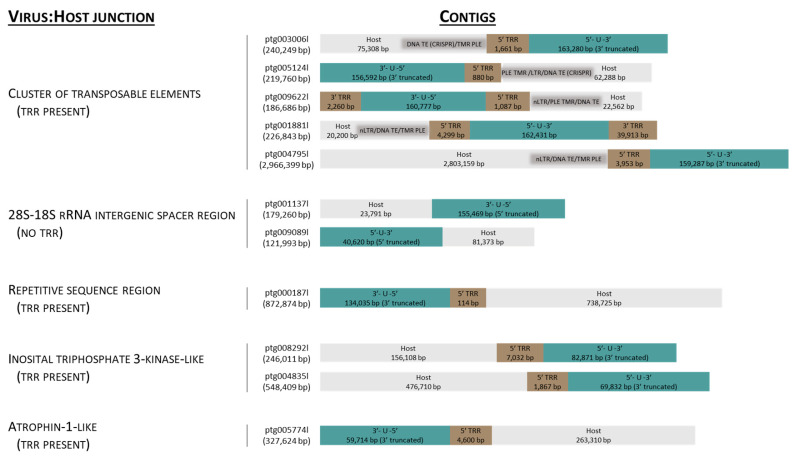
Schematics of contig sequences (5′–3′) illustrating the location of the acipenserid herpesvirus 3 (AciHV-3) genome relative to Lake Sturgeon *Acipenser fulvescens* chromosomal DNA. Features of the virus–host junction are provided on the left side of the figure. Contig id and length in base pairs (bps) are provided to the left of each rectangle in which the AciHV-3 genome unique (U) region is shown in blue, its terminal repeat region(s) (TRR) in brown and host sequence in grey. The dark grey box within the host sequence illustrates regions rich in transposable elements (TEs) including Penelope-like elements (PLEs), (non)long terminal repeat retrotransposons (nLTRs, LTRs) and various DNA TEs, some of which encode the CRISPR-associated protein Csa3 (CRISPR). The orientation and integrity of the U region is provided within each blue box. The number of base pairs within each colored box denotes the length of each region within the contig sequence. Additional details are provided in Table S3. Those contigs encoding TRR1 and TRR2 contain the complete virus genome. nt, nucleotide.

**Figure 3:**
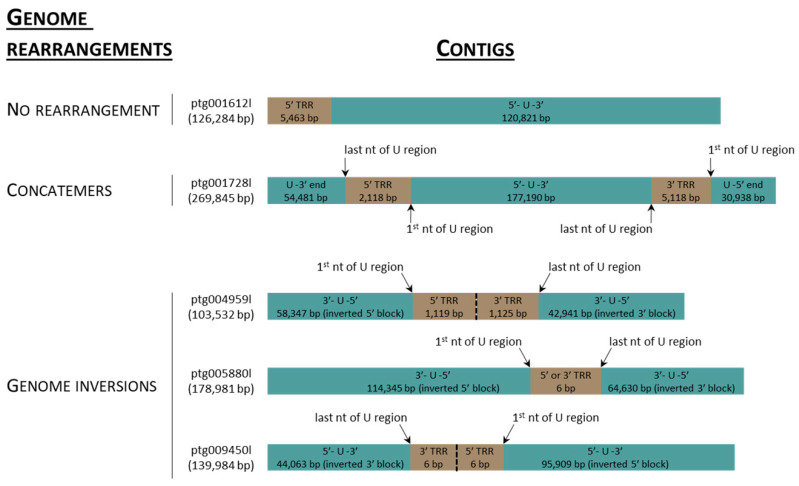
Schematics of contig sequences (5′–3’) in which the acipenserid herpesvirus 3 (AciHV-3) genome sequence is present in the absence of the host sequence. A description of the genome arrangement is provided on the left side of the figure. Contig id and length in base pairs (bps) are provided to the left of each rectangle in which the AciHV-3 genome unique (U) region is shown in blue and its terminal repeat region(s) (TRRs) in brown. The orientation and integrity of the U region is provided within each blue box. The number of base pairs within each colored box denotes the length of each region within the contig sequence. Additional details are provided in Table S3. nt, nucleotide.

Contigs (n = 16,219) longer than 10 kbp were assembled from 5.32 MHiFi reads. Of these, thirty contigs were found to contain >30 kbp AciHV-3 LSGO (Table S2) sequence. The sixteen longest contigs ranging in size from 103,532 to 2,966,399 bp were selected to characterize the genome sequence of AciHV-3 LSGO (Figure 2 and Figure 3). The coarse AciHV-3 LSGO genomic structural features present in these contigs are reported in Table S3, and the corresponding sequences are presented in Data S2.

### 3.2. Genome Structure and Composition

**Figure 4:**
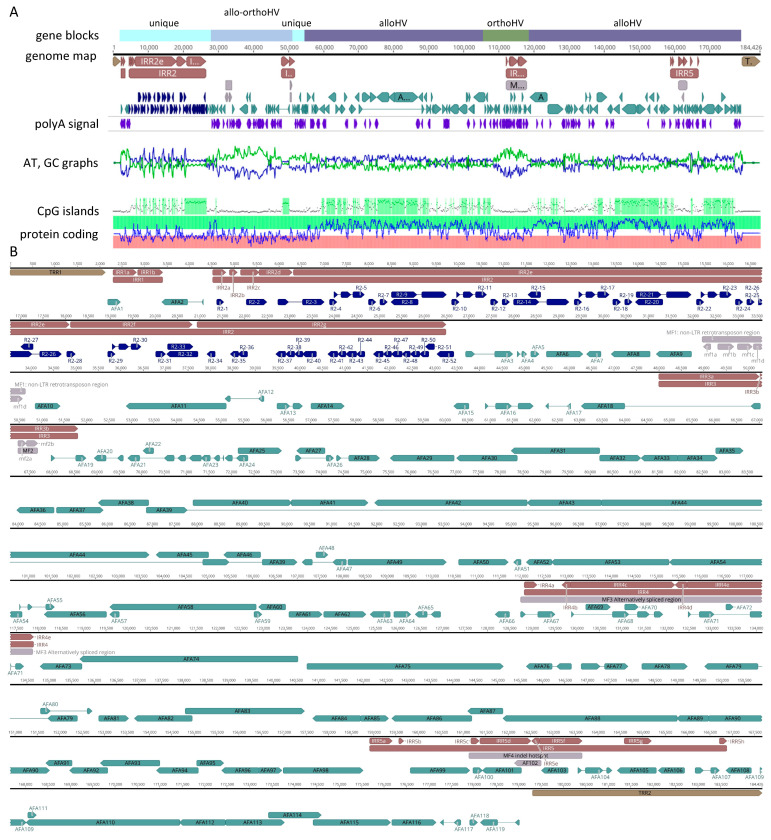
Genome map for acipenserid herpesvirus 3 from the Lake Sturgeon *Acipenser fulvescens* gonad cell line. The genome map represents an informed interpretation of bioinformatic-based predictions of ORFs, splice sites and repeat sequences. (**A**) Genome-wide mini-maps are presented for the gene blocks (*Alloherpesviridae* (alloHV), *Orthoherpesviridae* (orthoHV) or neither family (unique)), poly(A) signal sequence (i.e., aataaa), AT (green) or GC (blue) content of the nucleotide sequence, CpG islands and predicted protein coding regions. (**B**) Enlarged genome map in which the terminal repeat regions (TRR1 and 2; brown), internal repeat regions (IRR1 to 5; red), ORFs *R2-1* to *R2-52* in IRR2 (dark blue), unique region with predicted functional ORFs *AFA1* to *119* (blue) and miscellaneous features (MF1 to 4 and *AFA102*, grey) are presented for the 184,426 bp genome. Introns are displayed as a thin line between coding regions. Image created in Geneious v2023.2.

A complete AciHV-3 LSGO genome sequence was recovered from each of three contigs (ptg001728l, ptg001881l and ptg009622l) and has been reported based on nucleotides 54,482 to 238,907 of ptg001728l (Genbank accession number PQ564448). The genome was 184,426 bp in length and consisted of five IRRs (1105 to 21,971 bp) within a 177,190 bp U region flanked by terminal repeat regions (TRRs) of 2118 and 5118 bp (Figure 4). The length and composition of the repeat regions, and hence the genome, varied amongst the three contigs (Table S3). The GC content of the genome was 49.5% with CpG deficient areas of the U region displaying lower values ranging from 31.6 to 41.5% (Figure 4).

The putative first (nt 2119) and last (nt 179,308) nucleotides of the U region were identified by comparison of sequences from contigs containing either genome concatemers (ptg001728l), an inversion of sequence blocks within the genome (ptg005880l) or an integrated genome with no TRRs at the virus–host junction (ptg001137l, ptg009089l). The potential start of the U region was located immediately 3′ to TRR1 with sequence heterogeneity displayed in the first two nucleotides (TA, TT and CA) (Figure S2A). The U region appeared to end on the last G of a single TAGGG pentamer located immediately 5′ to TRR2. The proposed 5′ and 3′ termini of the U region also contained reverse complement counterparts of a 13–20 bp inverted repeat (Figure S2B). The length of the repeat depended on which 5′ terminal nucleotides were present with 5′-CA-3′ producing the longest perfect repeat (Figure S2B). Nucleotides of the 12 bp inverted repeat counterpart located at the 3′ terminal end of the U region were duplicated in two 44 bp tandem direct repeats (Figure S2C).

The major repeat regions consisted of dense clusters of simple, heterogenous (non)tandem direct repeats. Among the three contigs containing the full virus genome, the TRRs were comprised of perfect and imperfect telomere-like 6-mers arranged as tandem direct repeats with the arrays ranging in size from 1087 to 39,907 bp (Table S3 and Figure 2 and Figure 3). The AciHV-3 genome reported here contained 353–853 perfect copies of the hexamer TTAGGG. The smallest TRR was comprised of one hexamer located between inverted regions of the genome in ptg005880l (Figure 3). No TRR2 was evident at the 3′ end of the 5′ truncated genome present in ptg001137l and ptg009089l (Figure 2). Pac1- and pac2-like sequences were present in the genome (Table S4). Since none of them were located near the terminal ends of the genome, they were not considered likely candidates (Table S4). The first three IRRs were modal in size with all five IRRs present in the three contigs encoding the full virus genome. The largest and most complex internal repeat region, IRR2, varied in size (6686 to 21,971 bp) and repeat structure (largest direct repeat varied from 1147 to 6311 bp) amongst the three contigs (Table S3). The complex composition of the direct repeats in IRR2 to IRR5 of the AciHV-3 LSGO genome is illustrated in Figure S3. Short inverted repeat units (<100 nt; 89.7–100% identity) were found throughout the genome (Figure S3). In some cases, the inverted copies were located in different internal repeat regions: IRR2/IRR4, IRR2/IRR5 and IRR3/IRR5 (Figure S3). The longest of these units was 115 nt with inverted copies in IRR2a and IRR4d. The only other inverted repeat unit of notable length was comprised of 174–176 nt imperfectly matched units (95% identity) located in IRR5 (Figure S3).

### 3.3. Gene Arrangement

The genome annotation of the U region revealed at least 119 ORFs referred to as *AFA1* to *AFA119* (Table S5) and arranged as illustrated in Figure 4. A unique naming system was used for ORFs in IRR2 to account for the repeat heterogeneity that resulted in variable numbers of ORFs in this region across contigs (i.e., *R2-1* to *R2-52*).

**Figure 5:**
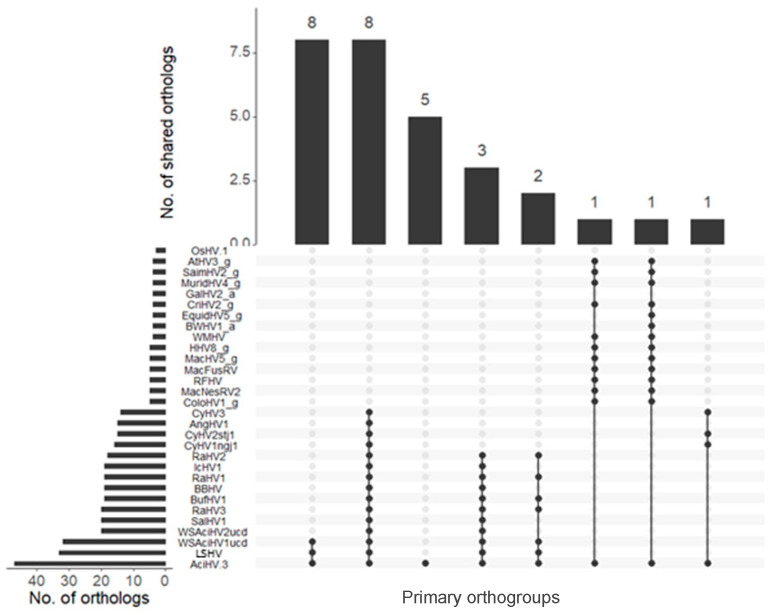
Orthology analyses of acipenserid herpesvirus 3 (AciHV-3). The UpSet plot displays the distribution of orthogroups between AciHV-3 and 29 members of the Herpesvirales order. The number of per-species orthologs are shown on the *x*-axis, whereas the shared orthologs are enumerated using vertical bars (annotated with number) for each orthogroup. The black dots represent the occurrence of a specific orthogroup in each species.

Orthology analysis based on 129 herpesvirus proteomes (n =11,296 genes, Data S1) grouped 87.4% of the viral genes into 1265 orthogroups, with 47 orthogroups containing AciHV-3 hits, for a total of 75 ORFs. The four AciHV-3 orthogroups with the most hits included DNA packaging terminases (n = 108 orthologs), ribonucleotide-reductases (n = 94), serine/threonine protein kinases (n = 62) and membrane proteins (n = 25). Five orthogroups exclusive to AciHV-3 were comprised of 23 ORFs that included the genes just described. For the 47 orthogroups with AciHV-3 hits, eight orthologs were shared by the AciHV-1-AciHV-3 group of sturgeon HVs, and a different set of eight orthologues were shared by representatives of the *Alloherpesviridae* family, including fish and ranid HVs (Figure 5). Interestingly, a cyclin D homolog was present in the genomes of AciHV-3 and several gammaHVs but not alloHVs (Figure 5).

The first 55 kbp of the genome contained a sequence block encoding *Alloherpesviridae* and/or *Orthoherpesviridae* (*Allo-Ortho*HV) homologues (nucleotides 28,728–50,911) flanked on either side by regions that encoded unknown or non-Herpesvirales sequences (nucleotides 2176–28,557 (except *AFA2*); 51,217–53,497). The *Allo-Ortho*HV block contained remnants of RTE-X and CR1 non-long terminal repeat (non-LTR) retrotransposons (nucleotides 32,200–33,869) including partial sequence homologues of CR1 reverse transcriptase and endonuclease/exonuclease/phosphatase proteins. Given the degraded condition of the ORFs, they are unlikely to encode functional proteins, which is why this cluster was annotated as a miscellaneous feature (i.e., MF1). The genes encoding the twelve core proteins of alloherpesviruses were intact and distributed in two sequence blocks (nucleotides 54,562–106,085 and 118,028–179,016) (Figure 4). These regions also encoded other homologues of proteins from LSHV and AciHV-1 UC Davis (n = 50 (including core proteins); Table S5). Although syntenic blocks of ORFs homologous to AciHV-1 *ORF20* to *26* (i.e., *AFA46* to *40*) and *ORF51* to *60* (i.e., *AFA86* to *95*) were identified, the overall gene arrangement within the AciHV-3 LSGO genome was unique. The two *Alloherpesviridae*-specific blocks were separated by a block of sequence (nucleotides 106,039–117,884) encoding proteins with similarity to those from members of the *Orthoherpesviridae* family (Table S5). Of the 119 annotated ORFs from the AciHV-3 LSGO genome, 69 displayed homology to proteins encoded by members of the Herpesvirales order. The remaining 50 ORFs were unique (n = 24) or displayed variable levels of homology to proteins from non-Herpesvirales viruses (n = 26) including a gene with the helix-loop-helix-zipper domain found in v-myc (retroviruses) and c-myc (eukaryotic) proto-oncogenes (*AFA65*).

ORFs were annotated in IRR2, IRR4 and IRR5 but not in IRR1 or IRR3 (Table S5). Despite the observed heterogeneity in IRR2, the same protein sequences were detected, but the number of sequence copies varied between the contigs. Redundancy was evident in ORFs annotated in IRR2, particularly in IRR2g of the virus genome. In this case, the amino acid sequences encoded by ORFs *R2-34* to *R2-51* were short and similar to the N-terminal sequence of *R2-32*. This redundancy would provide multiple potential start sites that could be spliced to *R2-32* should future mutations eliminate the ORF start codon. IRR2e consisted of 4 copies of a sequence that encoded 6 ORFs (i.e., n = 24 ORFs in IRR2e) (Figure 4 and Table S5). These proteins were predicted to be functional but did not display homology to protein sequences in the Genbank databases. In some cases, distant homology was predicted to HHV-4 EBNA-1 protein (HHblits probability 1.8–3.4) or to proteins from viruses outside of the Herpesvirales order (HHblits probability 1.5–11.7; Table S5). The plasticity of IRR2 was further illustrated in three contigs, ptg001612l, ptg004835l and ptg005774l, each of which contained an insertion between IRR2d and IRR2e that encoded a T-antigen-like protein not evident in any of the other contigs containing this portion of the genome (Data S2). The ORFs detected in IRR1 and IRR3a were not annotated given their low coding prediction potential, which suggested the sequences were not encoding functional proteins (Figure 4, see mini-map displaying relative protein coding potential for genome). In IRR3b, two fragments of a Bcl-2-like coding sequence were located in a region containing direct repeat units. The apparent remnants, referred to as mf2a and mf2b (collectively MF2), were also present in ptg001881l and ptg009662l and seemed unlikely to encode a functional protein (Figure 4). However, copies of eight repeat units spanning the 5′ end of MF2 were similarly located bounding the 5′ end of the gene encoding AFA10, a functional Bcl-2-like protein (Figure 4). One of the repeat units was in the region between the two Bcl-2 fragments mf2a and mf2b. IRR4 was located in the *Orthoherpesviridae*-specific block and encoded proteins homologous to vOX-2- or nectin-2-like immunoglobulin domain-containing proteins, specifically elephant endotheliotropic herpesviruses (subfamily *Betaherpesvirinae*) and human herpesvirus 8 (subfamily *Gammaherpesvirinae*) (Table S5). Analysis of predicted splice variants suggested that this cluster of ORFs could be differentially spliced together resulting in a diverse repertoire of proteins likely involved in immune response modulation. IRR5 appeared to contain sequence insertions and deletions and was hypervariable amongst the three contigs encoding the full virus genome (and other contigs encoding this region of the virus genome) (Table S5). For example, in IRR5 (1) the gene encoding AFA101 ranged in size from 573 to 873 nt, (2) the size of the *AFA102* gene varied from 219 to 591 nt and was annotated in the genome reported here as a miscellaneous feature since the coding sequence was truncated at 219 bp, (3) the *AFA105* gene was the site of at least 40 repeat units with ORF lengths varying from 399 to 1020 nt between contigs, and (4) all contigs had the 591 bp gene *AFA106*, but another ATG start codon was present in some contigs resulting in a longer ORF of 696 bp (Table S5).

Unusual genome arrangements including inversions or concatemers of the virus sequence were present in four contigs, none of which contained the host sequence (Figure 3). The contigs displaying inversions were similar in that the first and last nucleotide of the U region were now proximal to each other and separated by 1 (ptg005880l), 2 (ptg009450l) or 373 (ptg004959l) copies of the hexamer TTAGGG (Figure 3 and Table S3). The inversion in contig ptg005880l appeared to originate in IRR4. Since the genomes in the other two contigs were incomplete, the site where their inversion occurred could not be identified. Only one contig displayed a concatemeric-like arrangement of the genome (Figure 3; ptg001728l). In this case, the full genome was flanked by the last 54,481 bp of the genome on the 5′ end and the first 30,938 bp of the genome on the 3′ end (Figure 3 and Table S3).

### 3.4. AciHV-3 Genome Is Contiguous with Host DNA

The AciHV-3 LSGO genome was contiguous with Lake Sturgeon chromosomal DNA in contigs encoding both virus and host genomic regions (Figure 2, Table S3 and Data S3). Analysis revealed five patterns associated with the site of virus integration: (1) adjacent to a region encoding a cluster of transposable elements (TEs); (2) within the 28S–18S rRNA intergenic spacer region (ISR); (3) within a highly repetitive sequence homologous to chromosomal sequence from Sterlet Sturgeon; (4) next to a region encoding an inositol polyphosphate kinase-like protein; and (5) adjacent to a region encoding an atrophin-1-like protein (Figure 2 and Data S3).

A Penelope-like element (PLE) was a common feature of the host sequence in which the virus–host junction was adjacent to a cluster of transposable elements (Figure 2, Table S3 and Data S3 and S4). In these contigs, the virus genome was oriented such that the junction was bordering TRR1. With one exception (ptg009622l), the PLE was immediately adjacent to TRR1, and in all cases, the PLE was bounded on the other side by telomere-like repeats (TMRs) separating it from the other TEs (Figure 2). Otherwise, the organization of TEs appeared random within these clusters (Data S3). Both classes of TEs—class I, retrotransposons and class II, DNA transposons—were represented (Data S3 and S4). Although members of both subclasses of retrotransposons were identified, non-LTR retrotransposons (e.g., CR1, L2) were found more often than the LTR retrotransposons (e.g., Ty-3 gypsy) (Data S3). One notable DNA transposon of the IS630-Tc1-Mariner family encoded a transposase containing the CARF domain from Csa3, a protein of the Clustered Regularly Interspaced Palindromic Repeats (CRISPR)–CRISPR-associated (Cas) systems (e.g., ptg003006l, ptg005124l) (Data S3 and S4). The TE ORFs appeared to be truncated in some clusters, particularly in ptg009622l, which implied that not all host TE-associated coding sequences encoded a functional protein (Data S2, S3 and S4). The unique composition of each TE cluster suggested that the virus–host junctions found within this subset of contigs represented different integration sites.

The site of virus integration found in two contigs was within the host sequence identical to the Lake Sturgeon 28S–18S rRNA intergenic spacer region (Genbank accession number FJ688020.1) (Figure 2, Table S3, Figure S4 and Data S3). Insertion of the virus genome immediately 5′ to nucleotide 6415 of this host sequence was shared by contigs ptg001137l and ptg009089l. In both cases, the virus genome was oriented such that the 3′ end of the U region was contiguous with the host sequence (Figure 2). TRR2 was not evident. A single TTAGGG hexamer was present 5′ to the insertion site in the virus-negative sequence (Figure S4 and Data S3).

The virus integration site found on ptg00187l was unique amongst the contigs (Figure 2 and Table S3). In this case, TRR1 was adjacent to 738,725 bp of the host sequence containing a high density of heterogenous direct repeats (Data S3). The sequence was predicted to have low coding potential except for the 10,114 bp of DNA directly adjacent to the virus genome and a block encoding a non-LTR retrotransposon located 410,747 bp away from the integration site at nucleotides 544,896–548,282 of ptg00187l (Data S3). A BLASTN v2.13.0 to 2.16.0 search of the Genbank nr database with 3159 bp of the adjacent DNA revealed that the sequence was >87% identical to sequence from multiple chromosomes of the Sterlet Sturgeon genome (Data S3).

The 5′ end of the virus genome was also found to be contiguous with a region of the host sequence encoding an inositol-triphosphate 3-kinase C-like protein (IPK-like (pfam03770); ptg008292l, ptg004835l) (Figure 2, Table S3 and Data S3). In these contigs, 4846–4932 bp of DNA 87–88% identical to Sterlet Sturgeon chromosomal DNA separated TRR1 and the ORF encoding part of the IPK-like protein. The protein also contained conserved domains corresponding to the Rho transcription termination factor (PRK12678) and the CC1-like family of RNA splicing factors (TIGR01622) (Data S3). Two tandem copies of this 5 kbp block of sequence were present in ptg008292l.

The contig ptg0057774l contained a virus–host junction between TRR1 and a 13,081 bp region encoding small ORFs with no homologues in the NCBI Genbank nr database (Figure 2, Table S3 and Data S3). A BLASTN search of the database with the first 5686 bp of this region (nt 64,315–70,001) showed that regions of the sequence were >80% identical to Sterlet Sturgeon sequence from multiple chromosomes (Data S3). Immediately following the 13 kbp sequence block was DNA containing a high density of heterogenous direct repeats and encoding an 873 amino acid homologue of atrophin-1 (pfam03154), a protein associated with neurodegenerative disorders in humans (Data S3).

### 3.5. Genes Encoding Core Herpesvirus Proteins Are Intact

**Figure 6:**
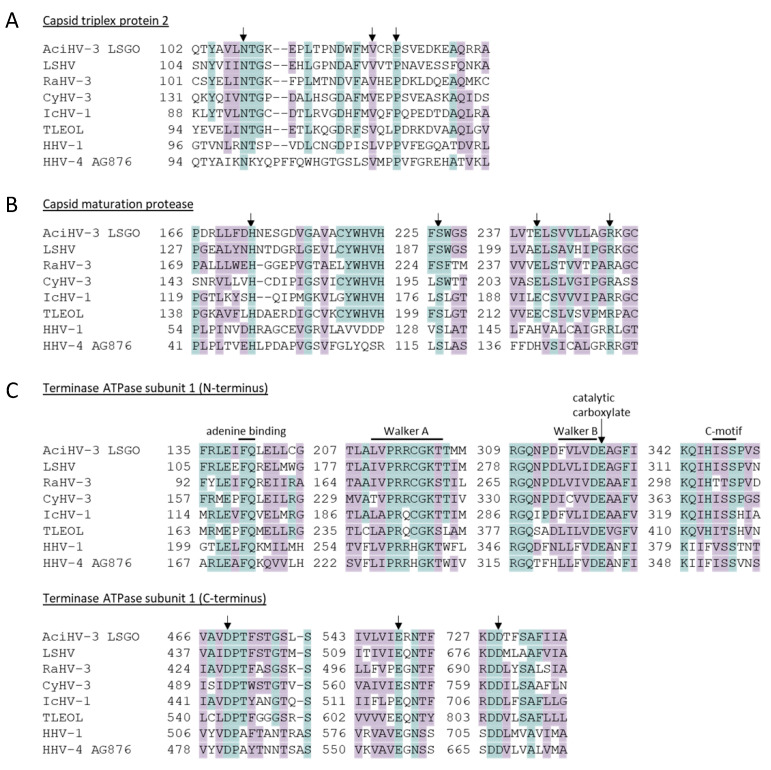
Location of conserved amino acids in universal Herpesvirales proteins involved in viral morphogenesis. The amino acid sequence alignments display functional signatures within the (**A**) capsid triplex protein 2 (critical amino acids involved in protein–protein interactions between capsid triplex proteins 1 and 2); (**B**) capsid maturation protease (key amino acids of the protease catalytic triad (H, S, E/H) and the oxyanion-binding site (R) conserved across the order Herpesvirales); (**C**) terminase ATPase subunit 1 (functionally significant amino acids within 4 regions of the N-terminal ATPase catalytic center and the catalytic metal-binding triad (D, E, D) of the C-terminal nuclease center). Conserved residues are highlighted in blue, and similar amino acids are highlighted in purple. Arrows show the location of conserved residues. The numbers correspond to the relative location of the first amino acid in the protein sequence that follows. The corresponding full protein sequence alignments are provided in Figure S5. Taxa are described in Table S2.

AciHV-3 orthologues of herpesvirus proteins involved in viral morphogenesis (capsid triplex protein 2, capsid maturation protease and terminase) contained key amino acids that are conserved across herpesviruses (Figure 6, Figure S5 and Data S5). For the capsid triplex protein 2, N^108^ and P^125^ of AFA116 corresponded to HSV-1 VP23 amino acids N^102^ and P^119^ that are conserved in triplex2 proteins from all members of the *Orthoherpesviridae* family, and V^122^ was similar to the orthoHV-conserved L^116^ (HSV-1 VP23) as both amino acids have hydrophobic side chains (Figure 6A) [70]. Other similar or conserved amino acids within this protein were observed within the N and C termini that have been shown to interact with triplex protein 1 (Figure S5) [70]. The AciHV-3 capsid maturation protease AFA98 contained the essential catalytic elements characteristic of serine proteases (i.e., H^173^, S^226^, E^240^), and also evident was R^249^, which provides the backbone amide of the oxyanion binding loop (Figure 6B and Figure S5) [72]. Within the AciHV-3 terminase protein AFA39, the conserved triad of amino acids (D^469^, E^548^ and D^729^) comprising the endonuclease active site was evident in the C terminus of the protein, whereas the conserved ATPase catalytic center comprised of four motifs (Walker A, Walker B, catalytic carboxylate and the ATPase coupling motif) was evident in the N-terminal domain (Figure 6C and Figure S5) [73,74,75].

A total of 48 nucleotide changes were identified in ORFs (28,896 nucleotides) encoding the 12 core alloherpesvirus proteins in contigs ptg001881l and ptg009622l relative to the AciHV-3 LSGO genome (Table 1 and Data S6). The majority of mutations were synonymous (64.5% (n = 31/48)) and no-nonsense mutations, insertions or deletions were found. Of the 17 nonsynonymous mutations, 10 resulted in conservative replacements, with amino acids having similar biochemical properties relative to their counterparts in the AciHV-3 genome (Table 1). If we use the number of non-conservative amino acid substitutions to derive a preliminary dN/dS ratio [87], the value 0.23 (7/31) is less than 1 and consistent with sequences evolving under purifying selection.

### 3.6. AciHV-3 LSGO Is Distinct from Teratorn-like Herpesviruses

**Figure 7:**
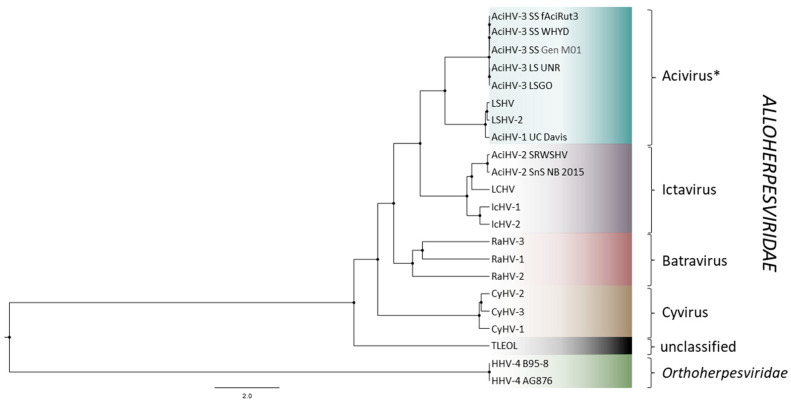
Bayesian phylogeny of concatenated amino acid sequences of 5 core alloherpesvirus proteins (capsid maturation protease, capsid triplex subunit 2, DNA polymerase catalytic subunit, helicase-primase helicase subunit and the major capsid protein). The tree was constructed under the LG amino acid substitution model [88] combined with a gamma-invariant site heterogeneity model with 4 rate categories, a strict clock and the Yule Process tree prior [89,90]. Full length protein sequences were used to generate the tree. Nodes are marked with a black circle of fixed size, and all node posterior probabilities are 1.0. The taxa are described in Table S2, and the *Alloherpesviridae* genera are provided to the right of the tree. * Acivirus is the genus name proposed in Clouthier et al. [25] for the unclassified AciHV-1 and AciHV-3 taxa.

AciHV-3 LSGO phylogeny was reconstructed using concatenated amino acid sequences of five core herpesvirus proteins from members of the *Alloherpesviridae* family (capsid maturation protease, capsid triplex subunit 2, DNA polymerase catalytic subunit, helicase-primase helicase subunit and the major capsid protein) (Figure 7 and Data S7). The AciHV-3 taxa clustered together in a strongly supported clade that shared a common ancestor with an apparent AciHV-1 clade comprised of AciHV-1 UC Davis, LSHV and LSHV-2 (posterior probability = 1). The tree topology was consistent with the AciHV-1 and AciHV-3 clades representing two new species that, together, appeared to comprise a new genus within the *Alloherpesviridae* family. The AciHV-1-AciHV-3 clade shared a strong ancestral node with the *Ictavirus* genus and was more distantly related to other alloherpesviruses including the Teratorn-like herpesviruses (Figure 7).

### 3.7. Q1mcp, a Sensitive Test for Specific Detection of AciHV-3 DNA

Q1mcp assay efficiency was consistent across different types of serially diluted templates with slope values ranging from −3.4 with plasmid or tissue DNA to −3.6 with cell lysate DNA (Figures S1 and S6, and Data S8). Linearity was high at 0.996 with plasmid DNA 10-fold serially diluted over nine orders of magnitude (10^0.7^ to 10^8.7^ copies). Addition of the artificial positive control probe did not change assay performance (linearity, 0.998; efficiency −3.5) (Figures S1 and S6). Collectively, these results suggested that the assay could be multiplexed and that plasmid DNA could be used to assess its performance. The quantity of tissue DNA added per reaction was typically 1500 ng and was not to exceed 4000 ng, above which interference of test performance was observed (Data S8).

**Figure 8:**
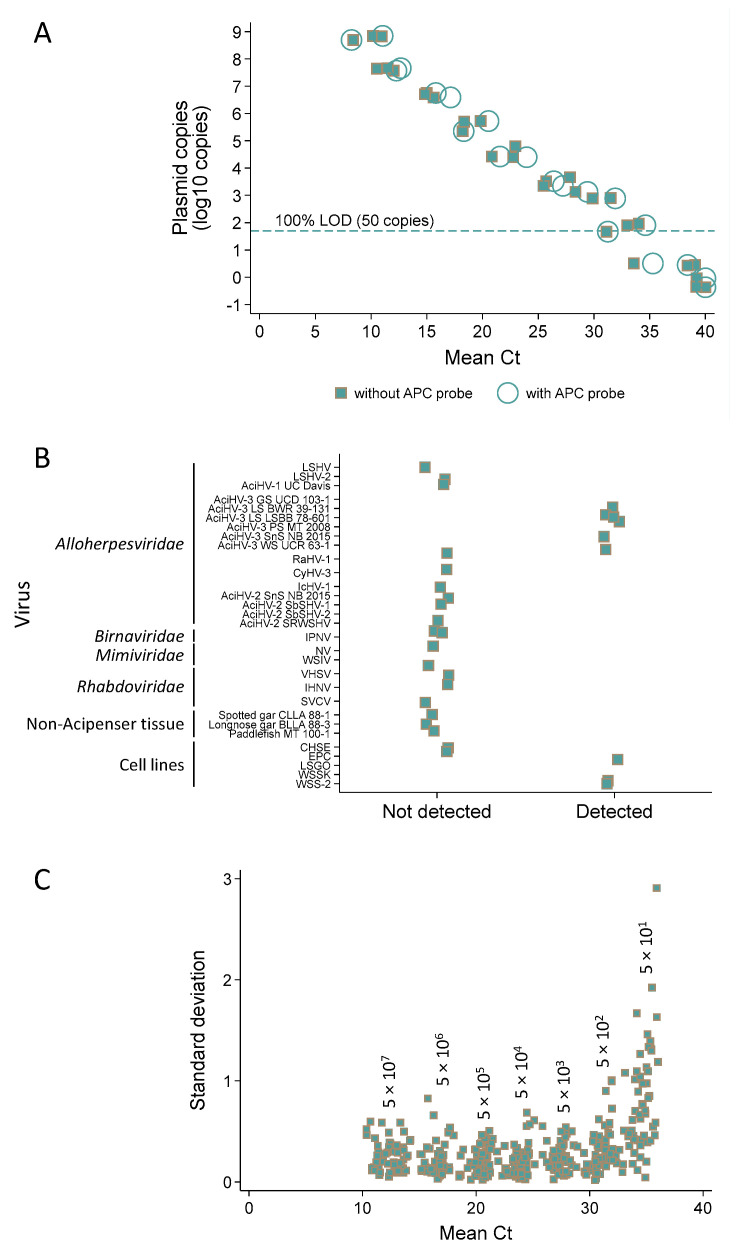
Analytical validation of the Q1mcp qPCR assay for detection of acipenserid herpesvirus 3 DNA. Unless noted otherwise, the assay was duplexed with the artificial positive control (APC) probe. (**A**) Analytical sensitivity—for each dilution, the mean cycle threshold (Ct) value for 3 replicates is reported for 2–3 runs of pLSAciHV3-APC plasmid DNA 10-fold serially diluted from 5 × 10^8^ to 0.5 copies. The dashed line marks the 100% limit of detection (LOD). Circles, with APC probe; squares, no APC probe. (**B**) Analytical specificity—binary test results of detected (<40 Ct) or not detected (40 Ct) with select viruses, tissues and naïve cell lines. (**C**) Analytical repeatability—for each dilution, the standard deviation and mean Ct value obtained for 3 replicates are plotted for pLSAciHV3-APC plasmid 10-fold serially diluted from 5 × 10^7^ to 50 copies (n = 58 runs).

Analytical sensitivity analysis revealed a 100% limit of detection at 50 copies for the single plex (nine of nine replicates tested positive) and multiplex assays (six of six replicates tested positive) (Figure 8A and Data S8).

The analytical specificity of the Q1mcp test (duplexed with the APC probe) was 100% inclusive (with 95% confidence intervals) of targets it should detect (100% (66–100)) and 100% exclusive of templates it should not detect (100% (84–100)) (Figure 8B; refer to Table S2 for description of targets, Data S8). Specifically, the assay detected AciHV-3 DNA present in other species of sturgeon and did not detect other viruses including AciHV-1 UC Davis, LSHV, LSHV-2 and AciHV-2 that may be sympatric with AciHV-3 in the same host. AciHV-3 DNA was detected in two Lake Sturgeon populations within Lake Superior (n = 10), whereas American Paddlefish from Montana (n = 10) and two species of wild gar from Louisiana (n = 10) tested negative. The results from taxa representing these populations are presented in Figure 8B.

The analytical repeatability of the Q1mcp test (duplexed with the APC probe) was evaluated using the results from 58 runs in which the artificial positive control plasmid pLSAciHV3-APC-Q1mcp was 10-fold serially diluted from 10^7.7^ to 10^1.7^ copies (three replicates per dilution) (Figure 8C, Table S6 and Data S8). Estimates of variability expressed as coefficient of variation values were generated from the Ct values obtained for each replicate. The intra-assay CV for Ct values ranged from 0.85 to 2.22 while the inter-assay CV values were between 2.48 and 8.35. In both cases, a larger variation was observed for samples containing the lowest and highest plasmid copy numbers (Figure 8C and Table S6).

### 3.8. AciHV-3 DNA Is Detected in High Titers in Somatic and Germ Line Tissues

**Figure 9:**
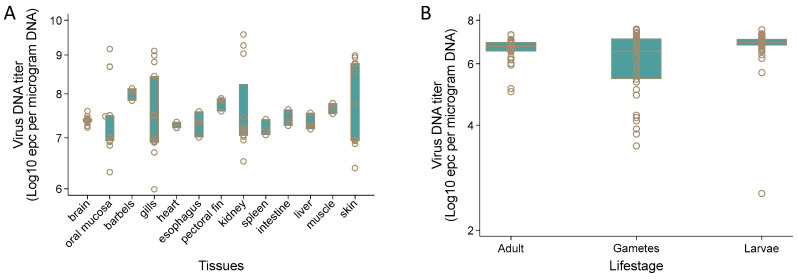
Acipenserid herpesvirus 3 DNA titer in tissues from Lake Sturgeon *Acipenser fulvescens* at different life stages. Virus DNA titer was determined using the Q1mcp qPCR test and expressed as equivalent plasmid copies (epcs) per µg DNA for (**A**) somatic tissues collected from juvenile Landing River Lake Sturgeon (age—0, 2017 year class (n = 10); age—1, 2019 year class (n = 3)) housed at the Grand Rapids Hatchery in Manitoba and (**B**) adult fin tissue, gametes (eggs, milt) and larvae (whole; 46–74 days post-fertilization) from select mating crosses of wild Lake Sturgeon broodstock from the Winnipeg, Landing or Burntwood Rivers. Further details are available in Table 2. The horizontal line within each box is the median virus DNA titer, the individual boxes represent 50% of the observations and the circles denote virus DNA titer values.

AciHV-3 DNA was detected by Q1mcp in 13 different somatic tissue samples from juvenile Lake Sturgeon (n = 3 to 13) including brain, oral mucosa, barbels, gills, heart, esophagus, pectoral fin, kidney, spleen, intestine, liver, muscle and skin (Figure 9A and Data S9). The mean DNA titer across all tissues was 10^7.52^ ± 10^0.75^ epcs/µg DNA. The highest value for the mean DNA titer by tissue was obtained with barbels (10^8.00^ ± 10^0.15^ epcs/µg DNA) and the tissue with the lowest DNA titer was spleen (10^7.21^ ± 10^0.18^ epcs/µg DNA). The difference between these values was statistically significant using the independent two sample *t*-test allowing for unequal variances (*p* < 0.05).

Pectoral fin tissue from 38 spawning wild Lake Sturgeon (n = 17 male, n = 21 female), their gametes (n = 17 milt, n = 21 egg samples) and larvae (n = 60 at 46–74 dpf) from select mating crosses tested positive by Q1mcp except for 2 progeny of the 2012 Winnipeg River broodstock (Figure 9B, Table 2 and Data S9). Since all DNA samples from tissues were normalized to ≤1500 ng prior to analysis by the qPCR assay and the positive control samples used for this set of tissue samples produced the expected results, these two negative findings were not considered to be false negative results. The same results were obtained upon repeat testing of these two samples. The mean virus DNA titers were 10^6.63^ ± 10^0.49^ (adult sturgeon), 10^6.12^ ± 10^1.14^ (gametes) and 10^6.83^ ± 10^0.64^ (larvae) epcs/µg DNA. The difference in virus DNA titer between the larvae with the highest mean titer and the gametes with the lowest mean titer was significant (unequal t-test *p* > 0.05). No difference in virus DNA titer was observed between the egg (10^6.15^ ± 10^0.18^ epcs/µg DNA) and milt (10^6.64^ ± 10^0.71^ epcs/µg DNA) (unequal t-test *p* > 0.05) samples. The individual sample with the highest virus DNA titer was an egg sample (10^7.54^ epcs/µg DNA) and one of the larvae displayed the lowest titer (10^2.55^ epcs/µg DNA).

### 3.9. AciHV-3 DNA Is Present in Lake Sturgeon Populations from Two Drainage Basins in Canada

**Figure 10:**
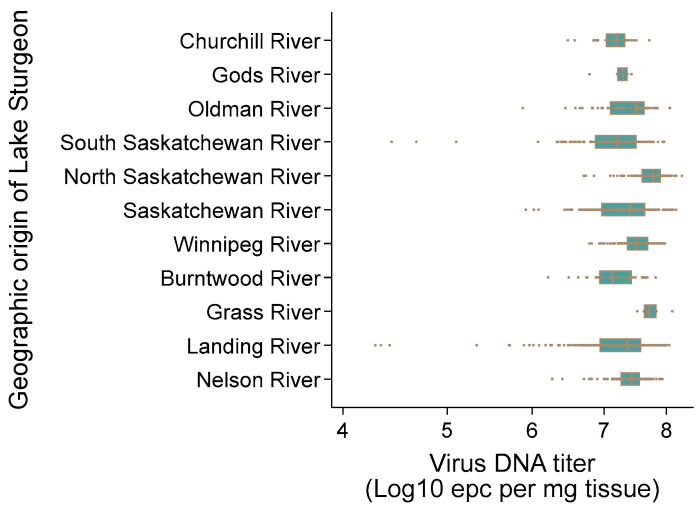
Acipenserid herpesvirus 3 DNA titer in fin tissue of wild Lake Sturgeon *Acipenser fulvescens* in the Hudson Bay drainage basin. The DNA titer was quantified as equivalent plasmid copies (epcs) per mg tissue using the Q1mcp qPCR test and the standard curve method. The range of values obtained for each population is presented according to their river of origin. The vertical line within each box is the median virus DNA titer, the individual boxes represent 50% of the observations, and the circles denote virus DNA titer values.

AciHV-3 DNA was detected by Q1mcp in 100% of the apparently healthy wild Lake Sturgeon (n = 1162) sampled from 10 rivers in the Hudson Bay drainage basin between 2010 and 2021 (Figure 1, Table S1 and Data S9). Non-lethal sampling was used, and only a subsample of the pectoral fin tissue was collected from these fish. The mean virus DNA titer across all populations was 10^7.34^ ± 10^0.46^ epcs/mg fin tissue (Figure 10). The highest mean virus DNA titer by river was obtained with the North Saskatchewan River population in Alberta, Canada (10^7.3^ ± 10^0.28^ epcs/mg tissue), and the South Saskatchewan River population from the same province had the lowest mean titer (10^7.1^ ± 10^0.6^ epcs/mg tissue) (Figure 10). The difference between these values was statistically significant using the independent two sample t-test allowing for unequal variances (*p* < 0.05). Individual sturgeon with the two lowest virus DNA titers were from the Landing River at the confluence with the Nelson River (10^4.28^, 10^4.33^ epcs/mg tissue), whereas the two sturgeon with the highest titers were from the North Saskatchewan River (10^8.27^, 10^8.17^ epcs/mg tissue) (Figure 10).

AciHV-3 DNA was also detected in 100% of the fins tested from 10 asymptomatic wild Lake Sturgeon from Batchawana Bay and Goulais Bay of Lake Superior within the Atlantic drainage basin (ADB) (Figure 1 inset). The mean virus DNA titer for both populations was 10^6.41^ ± 10^0.52^ epcs per mg of tissue (Data S9).

### 3.10. A New Genotype of AciHV-3 Is Detected in Green Sturgeon Acipenser Medirostris

**Figure 11:**
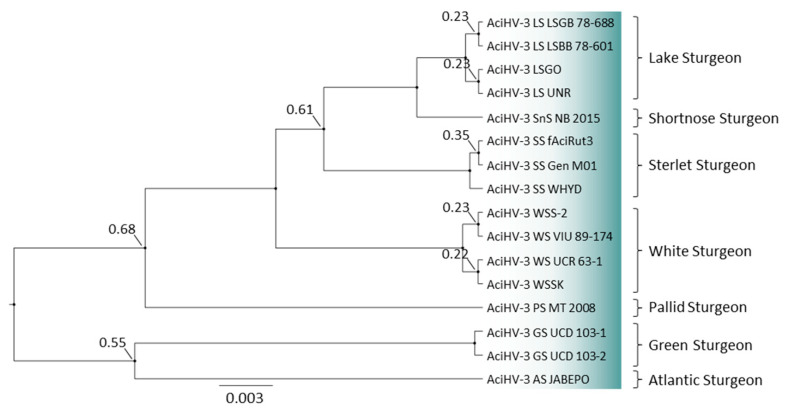
Phylogenetic tree of acipenserid herpesvirus 3 genotypes. A 493 bp sequence of the major capsid protein gene from each sample was aligned in ClustalX2 and the Bayesian inference tree was reconstructed under the HKY model [91]. Ancestral nodes are fixed in size and labelled if the posterior probability of their existence was <0.997. Taxa are described in Table S2, and their host of origin is provided to the right of the tree.

Bayesian phylogeny revealed that Lake Sturgeon AciHV-3 from the two drainage basins (Hudson Bay drainage basin (HBDB) and Atlantic drainage basin (ADB)) clustered together with AciHV-3 LSGO in a monophyletic lineage (Figure 11 and Data S10). The analysis was performed with the partial mcp sequence amplified using the genotyping GCmcp test. DNA sequence identical to the genotyping fragment from AciHV-3 LSGO was generated with DNA from the ten Hudson Bay drainage basin samples (one per river; represented by AciHV-3 LS UNR) as well as DNA from the two Atlantic drainage basin samples (AciHV-3 LS LSGB 78–688, AciHV-3 LS LSBB 78–601). The Lake Sturgeon clade shared a strongly supported common ancestor with AciHV-3 from Shortnose Sturgeon (Figure 11). The phylogeny was reconstructed with AciHV-3 from other sturgeon species including two new sequences from Green Sturgeon (i.e., AciHV-3 GS UCD 103-1 and 103-2). In this case, the genotyping sequences obtained from mucus samples were identical to each other and contained nineteen single nucleotide polymorphisms (SNPs) relative to the Lake Sturgeon AciHV-3 sequence. The Green Sturgeon AciHV-3 clustered together in a strongly supported clade and shared a poorly resolved common ancestor with Atlantic Sturgeon (Figure 11).

## 4. Discussion

Understanding the biological consequences of viral genome integration in carriers of ciAciHV-3 will be important to the future development of effective management strategies for wild and cultured sturgeon species, especially those that are threatened or endangered. In this study, we present the genome sequence of the alloherpesvirus AciHV-3 LSGO and show that the putative virus has lifecycle features similar to other chromosomally integrated herpesviruses including iciHHV-6 in humans. Long read DNA sequencing results show that the AciHV-3 genome is contiguous with *A. fulvescens* DNA and support our hypothesis that Lake Sturgeon carry one or more copies of the integrated viral genome in their chromosomal DNA. The antiquity of the original integration event is inferred by the presence of AciHV-3 DNA in six other sturgeon species whose biogeographic interaction predates sturgeon speciation. Despite this apparent ancient origin, the virus genome appears to encode key *Herpesvirales* ORFs with intact open reading frames and functional signatures as well as mutation profiles that are dominated by synonymous changes. Additional studies are required, but these features provide preliminary evidence that the virus genome is evolving under purifying selection, a condition that is reminiscent of a replicating virus. Phylogenetic analysis conducted with core herpesvirus proteins confirms our previous findings [25] that AciHV-3 is an alloherpesvirus and as such is the second lineage within this family, aside from the Teratorn-like herpesviruses [92], to display an endogenous lifestyle. Although both virus lineages are members of the same family, tree topology reveals a distant evolutionary relationship. qPCR testing established the ubiquitous presence and high titer of AciHV-3 DNA in both somatic and germ line tissues and provided evidence that, at some point, integration of the AciHV-3 genome occurred in the chromosome(s) of Lake Sturgeon germ cells. The presence of AciHV-3 DNA in 97% of the larvae tested from select mating crosses supports a Mendelian pattern of virus inheritance. In this case, the qPCR diagnostic and phylogeographic results reveal that the virus genome has reached genetic fixation given its endemicity at 100% prevalence in Lake Sturgeon across the Hudson Bay drainage basin. Together, these results are consistent with an alloherpesvirus that has established a latent infection through chromosomal integration of the virus genome in germ line cells, is inherited by vertical transmission of the genome as a host allele and is also capable of reactivating to become a replication competent virus with the capacity to infect new hosts via horizontal transmission pathways.

The AciHV-3 LSGO genome map revealed a 177 kbp U region containing 5 IRRs and flanked by terminal direct repeats consisting of perfect TTAGGG repeats reminiscent of the TMRs present in all vertebrates [93]. The location of TMR arrays between adjacent head to tail copies of the AciHV-3 LSGO genome in contig ptg001782l was interpreted as strong evidence that they were part of the virus genome rather than host-derived sequence. The first and last nucleotides of the U region were assigned to the respective residues immediately 3′ to the last hexamer of TRR1 and 5′ to the first hexamer of TRR2. The presence of TMRs in a herpesvirus genome is not unique to AciHV-3 since they have been detected in other alloHVs (e.g., CyHV-1, CyHV-2 and CyHV-3) as well as orthoHVs (e.g., HHV-6 and MDV) [94]. Similar to the AciHV-3 LSGO genome, their TMRs are located at their genome termini. Although the arrangement of terminal TMR arrays flanking a U region was replicated in all three contigs encoding the full AciHV-3 LSGO genome, heterogeneity was observed in the relative length of the terminal and internal repeat regions. These differences produced variability in genome size with the total length ranging from 164,124 to 206,643 bp and the corresponding U regions varying between 160,777 and 177,190 bp. Despite this plasticity, ORF arrangement and coding capacity were conserved between the three genomes. The genome arrangement of AciHV-3 is not strictly characteristic of any of the known Herpesvirales members [3], but its internal repeat regions resembled those found on the class C genome of EBV. The absence of any apparent genetic relationship between AciHV-3 and EBV or other lymphocryptoviruses implies that this genome structure has evolved independently in HV lineages infecting mammals and fish and reflects the lack of correlation between genome structure and evolutionary relatedness noted for other herpesviruses [3,95].

An unusual feature of the AciHV-3 genome was the presence of an orthoherpesvirus-like gene block separating two *Alloherpesviridae*-specific regions. BLAST analyses with protein sequences encoded by the orthoHV block revealed strongly supported hits to homologues from sturgeon species as well as beta- or gammaHVs. In general, stronger support was obtained for the sturgeon homologues relative to the orthoHV homologues. Gene capture of cellular homologues through horizontal gene transfer is presumed to have occurred throughout herpesvirus evolution [3]. However, herpesviruses have also acquired genes (e.g., rep gene in HHV-6) from other DNA viruses (e.g., adenoviruses), and remnants of LTRs have been found in alphaHV genomes [96]. The latter is similar to our finding of mutated non-LTR retrotransposon sequences in the AciHV-3 genome and may be indicative of a potential mechanism of gene capture in herpesviruses. Whether the genes within the AciHV-3 orthoHV block have cellular or viral origins (or some combination of both) is unknown, but acquisition of foreign genomic fragments by this virus may be facilitated by its apparent endogenous/exogenous lifestyle.

The new genetic data from AciHV-3 enabled the design of the Q1mcp qPCR assay whose analytical performance was validated according to international standards [97]. The test is pan-specific for AciHV-3 sequences found in North America including the more divergent Green Sturgeon lineage with nineteen SNPs in the cPCR genotyping product. The assay was exclusive of other viruses that have been found in sturgeon including AciHV-1 UC Davis, LSHV, LSHV-2 and AciHV-2. This finding is consistent with the low amino acid sequence similarity found between the AciHV-3 mcp and its homologues from AciHV-1 (48% similarity) and AciHV-2 (43% similarity) [25]. The assay’s linearity and efficiency remained high even when its performance was evaluated over nine orders of magnitude. Assay design enabled detection of false positive results that could arise from positive control samples that were included at each step of the sample processing from nucleic acid extraction to qPCR. These performance features as well as the high repeatability of the assay evident from the validation study provided confidence in the qPCR results generated in this study.

The continuity of AciHV-3 DNA with host chromosomal DNA in conjunction with the high virus DNA titers and the ubiquitous presence of AciHV-3 DNA in somatic and germ line tissues suggest that integration of the virus genome has occurred in host chromosomes, specifically those of germ cells. Germline integration of a virus genome would mean that at least one copy of that genome is present in every nucleated cell of the body [14], a condition that would explain the high virus titer (i.e., DNAemia) and ubiquitous presence of AciHV-3 DNA within thirteen different somatic tissues. Although a causal relationship has not been established, we suspect that future work will identify a link between ciAciHV-3 and the herpesvirus-like epithelial skin lesions previously observed in juvenile Lake Sturgeon, especially since no other etiological agent has been identified through NGS [25]. If we consider this possibility here, then it is notable that pectoral fins were among the tissues with the highest viral DNA titer and were one of the few places where lesions were observed on symptomatic sturgeon. The variability in DNA titer observed across tissues may be related to localized replication of exogenous AciHV-3, whereas the inter-fish variability may be due to differences in the number of chromosomes (ploidy) and/or the number of virus integration events per host genome. The unusual characteristics of AciHV-3 mimic the hallmark features of iciHHV-6, the only extant germline integrated herpesvirus [14]. This virus is known to integrate into the sub-telomere regions of human chromosomes [10,98].

Only general statements can be made from this study about the integration sites of the ciAciHV-3 LSGO genome since a chromosome-level assembly of the Lake Sturgeon genome is not available. A total of ten different AciHV-3 LSGO genome–host junctions were identified suggesting insertion site polymorphism. This pattern is similar to the heterogeneity of integration profiles observed for other endogenous herpesviruses including MDV [9,99,100], EBV [101,102] and HHV-6 [14]. The genomes of these orthoHVs have terminal telomeric repeat arrays that are similar to the telomere-like sequences present in the AciHV-3 genome [94]. In fact, these repeats have been found to be critical for the integration and maintenance of MDV and HHV-6 genomes in the telomeres of host chromosomes [10,98,100,103,104,105,106].

The telomere-like terminal repeat regions of the AciHV-3 genome were present in nine of our contigs, a subset (n = 5) of which displayed genome insertion sites located adjacent to degraded Penelope-like elements containing a GIY-YIG endonuclease domain. PLEs of this type integrate at random sites within chromosomes, preferring AT-rich regions [107]. Although these PLEs are not part of the AciHV-3 genome and did not provide insight into the virus integration site, this co-location of (retro)transposon-rich regions with a herpesvirus genome is reminiscent of the TE–HV fusion that resulted in the Teratorn mobile element [92]. Unlike the active transposition activity of Teratorns [92], the PLE retrotransposons do not appear to be functional since the canonical PLE ORF was not intact. Notably, the PLE sequence was bounded by a TMR-like sequence of variable length which may have promoted integration of the AciHV-3 genome at these sites.

The integration site that was most informative as to the general location of the virus genome in Lake Sturgeon chromosomes was the site within the 28S–18S rRNA ISR (ptg001137l and ptg009089l). Studies by Fontana et al. [108,109] revealed that the 28S–18S rRNA genes of six sturgeon species were localized to the telomeric regions of multiple chromosomes using fluorescent in situ hybridization techniques. Although Lake Sturgeon was not one of the species evaluated, the pattern was conserved across species and is likely to apply to Lake Sturgeon. If so, then AciHV-3 appears to mimic the telomere integration of HHV-6 and MDV. We speculate that the AciHV-3 genome integrates into host telomeres by homologous recombination between the host telomeres and the virus TRRs, similar to the process documented for MDV [103] and HHV-6 [13,98,106]. As proposed for iciHHV-6, the integration event may occur during the repair of single- or double-stranded breaks in the host DNA at or near the telomere/subtelomere region [14]. Both pathways would involve invasion of the host genome via the homologous host telomeres and viral TMR followed by extension of the viral TMR to restore functional host telomeres [14]. The proximity of the AciHV-3 genome integration site to clusters of transposable elements suggests that, in some cases, the virus genome may have fortuitously integrated at breaks in the host DNA caused by these mobile genetic elements, seemingly favoring those associated with Penelope-like elements. Unlike humans whose telomeric repeats are located at the terminal ends of each chromosome [110], sturgeon have telomere DNA repeats interspersed along the entire length of some of their microchromosomes [111]. As a result, the location of AciHV-3 genome integration may not be confined to the terminal ends of chromosomes, a feature that will have to be considered when interpreting future images of chromosome spreads hybridized with fluorescently labelled AciHV-3-specific probes. Telomeric TRRs were not evident in the two AciHV-3 genomes integrated within the ISR, but it is interesting that a single TTAGGG hexamer is located immediately 5′ to the insertion site in the virus-negative sequence (Figure S4) and that these two contigs were the only ones in which the virus–host interface occurred near the 3′ end of the virus. Perhaps one TTAGGG unit in the host DNA is enough for recombination with the virus genome to occur followed by integration. A chromosomal-level assembly of the Lake Sturgeon genome will be instrumental to understanding more about the distribution of ciAciHV-3 within the host chromosomes.

The intact coding capacity of genes encoding core alloherpesvirus proteins and the presence of functional signatures in core proteins involved in virion morphogenesis provided evidence that AciHV-3 has been evolving under continuous purifying selection and hence, in principle, appears to have retained the capacity to be a fully functional exogenous virus despite the apparent antiquity of the original integration event. Additional work will be required to confirm that AciHV-3 core genes encode biologically functional proteins; however, these preliminary results, in conjunction with the existence of contigs encoding rearrangements or concatemers of the AciHV-3 genome, support our hypothesis that ciAciHV-3 has the potential to reactivate and produce virions. The presence of concatemers of the AciHV-3 genome is consistent with previous findings in which herpesvirus DNA circularizes and replicates via rolling circle replication [5], and during a lytic infection, long concatemers of the genome are produced and subsequently cleaved at the time of packaging [112,113]. A recombination event occurring through the terminal repeat sequences could initiate AciHV-3 genome circularization, a process that may also involve the 12–20 bp inverted repeat found at the terminal ends of the U region. This event would be among the initial steps leading to the development of replication competent virions.

AciHV-3 LSGO replication would require excision of the integrated virus genome from the chromosome and reactivation as an exogenous virus. This cycling between endogenization as a form of latency and excision from chromosomes has been reported for iciHHV-6A [114] and iciHHV-6B [115]. These events could have deleterious consequences for the host such as destabilization of telomeres [116,117] as well as intermittent inflammatory or autoimmune responses [118]. Despite this, iciHHV-6 is present in 1–2% of the human population, and more striking is the 100% prevalence of AciHV-3 DNA in Lake Sturgeon of the HBDB. Endogenous virus elements that introduce neutral or mildly harmful polymorphisms may achieve fixation but will accumulate mutations at the neutral rate of the host [119,120]. In contrast, the genome of ciAciHV-3 appears to have retained functionality, thereby mimicking iciHHV-6. Genetic fixation of the ciAciHV-3 genome in the HBDB populations of Lake Sturgeon (and likely all sturgeon species) implies that the presence of the large virus genome does not impose deleterious consequences upon the host. AciHV-3 DNA was initially discovered in hatchery-reared juvenile Lake Sturgeon displaying epithelial skin lesions [25]. While the gross pathology and histopathological changes associated with the lesions were consistent with other herpesvirus infections, virions were not detected by electron microscopy [25]. If future studies provide evidence that ciAciHV-3 can indeed reactivate to an exogenous virus, then perhaps this potential endogenous/exogenous biphasic lifecycle confers selective advantages on both the virus and the host.

The proposed endogenization and excision of ciAciHV-3 in Lake Sturgeon genomes may also provide a mechanism that ensures a lifelong association of ciAciHV-3 with its host and enables persistence and maintenance of the virus genome in sturgeon populations. Once an endogenous virus such as ciAciHV-3 has reached genetic fixation in a host, the virus genome is inherited by all descendants of that host lineage. This expected pattern of genetic heritability was observed in our study with the exception of two larvae that tested negative by Q1mcp despite detection of AciHV-3 DNA in their parental germline tissues. A potential explanation is that the virus genome was lost during spontaneous changes occurring within the genomes of the two larvae. These changes can include the loss of entire chromosomes occurring as a result of the natural ploidy plasticity characteristic of sturgeon [22,121,122]. If the ciAciHV-3 genome proves to be fully functional and capable of reactivation to an actively replicating exogenous virus, then horizontal transmission of AciHV-3 shed from sympatric sturgeon would provide a pathway for ensuring both individual and population level protection and virus maintenance as well as opportunities for future somatic or germline integration events. This lifecycle would require host and/or virus-mediated processes for regulating expression of the integrated virus genome to ensure a transient and focal infection in select differentiated cells. The process may involve epigenetic modulation of virus genome transcription through DNA methylation and/or histone modification similar to that observed with endogenous retroviruses [123] and herpesviruses [124,125,126]. The presence of CpG islands in the AciHV-3 genome suggests that DNA methylation may transiently render parts of the genome transcriptionally inactive and immune masked. The trigger controlling the switch between an endogenous latent phase and the exogenous replicating phase may be environmental stimuli such as changes in water temperatures or alterations in host condition (e.g., spawning). These types of effectors have been associated with aquatic animal disease outbreaks caused by other epitheliotropic herpesviruses [127,128,129,130,131]. The epidemiological investigation that led to the discovery of AciHV-3 DNA revealed that the epithelial skin lesions on the abdomen of symptomatic fish were transient and eventually receded with no apparent negative effect on the host [25]. Histopathological changes were not observed in any other tissues from these fish [25] despite our detection of AciHV-3 DNA in all somatic and germline tissues tested in this study. Although unproven, if we speculate that the ciAciHV-3 genome is capable of reactivation to an actively replicating virus, then studies into a potential mechanism that selectively controls virus replication in epithelial cells of the skin may lead to further insights into the biology of the ciAciHV-3 genome.

The host-specific adaptation of AciHV-3 in sturgeon first reported in Clouthier et al. [25] is evident in the topology of our genotyping tree that includes new results from Green Sturgeon. The data continue to support our hypothesis that this alloHV lineage is orthologous across all sturgeon species. Negative qPCR test results with fin tissue from gar and paddlefish indicate that the virus genome is not present in the last common ancestor of the *Acipenseridae* and *Polyodontidae* families and that its host range may be restricted to members of the *Acipenseridae* family. These results will be useful for characterizing the paleovirological profile of this virus including when it emerged and/or integrated into the host genome. If we assume that this apparent host-specific adaptation is due to the more recent biogeographic isolation of extant sturgeon species as hypothesized in Clouthier et al. (2023) [25], then some genetic diversity might be expected in AciHV-3 DNA from disjunctive Lake Sturgeon populations that are separated by drainage divides (i.e., HBDB: DU1 to DU3 versus Great Lakes: DU4). Instead, the results reveal that Lake Sturgeon from both drainage basins carry the same virus genotype. These sturgeon populations descend from two different post-glacial sources with the HBDB sturgeon originating from the Missourian refugium and those from the Great Lakes are founded from a Mississippian source [132]. Since these Lake Sturgeon populations are considered genetically distinct and biogeographically isolated [24], the results suggest an evolutionary continuity of the AciHV-3 lineage that predates Lake Sturgeon occupation of either drainage basin. The lack of genetic diversity in AciHV-3 DNA between drainage basins also underscores the slow rate of evolution and/or the low intra-species genetic diversity in AciHV-3 sequence arising from genetic adaptations made to the same host species regardless of the environment. This apparent evolutionary stasis within the major capsid protein suggests a long-term association between this lineage of AciHV-3 and Lake Sturgeon. These findings are consistent with co-evolution of ciAciHV-3 and Lake Sturgeon similar to the virus–host phylogenetic congruence observed with other herpesviruses [2]. The co-evolution of AciHV-3 lineages in other sturgeon species is reflected in their host-specific clustering as monophyletic groups in the genotyping tree. If the virus genome is indeed present in all sturgeon species, then the virus likely emerged prior to sturgeon radiation.

An ancestral integration of the AciHV-3 genome occurring after divergence of sturgeon and paddlefish but prior to sturgeon speciation would explain the presence of the virus DNA in all sturgeon species analyzed to date (and its absence from paddlefish). The slow rate of ciAciHV-3 evolution is consistent with its endogenous lifestyle in which the virus genome appears to be integrated into a germline chromosome. Since the ciAciHV-3 genome has reached fixation as a genetic trait—at least in the Lake Sturgeon populations of central Canada—then the virus genome would be evolving at the host rate of evolution under the purview of a DNA polymerase with high fidelity proof-reading activity. If the ciAciHV-3 lineages expanded primarily through intra-host reproduction and vertical transmission (i.e., heritability), as proposed in Clouthier et al. [25] and evidenced here, then the ancestral integration event would eventually lead to the low inter-host virus sequence diversity observed for extant AciHV-3 lineages. Periodic imbalances in the benign and asymptomatic host–virus relationship could result in excision and horizontal transmission of the actively replicating virus followed by new integration events. Recent insertions of the virus genome arising from this proposed endogenous–exogenous cycle may explain the lack of mutations in the ciAciHV-3 LGSO genome as evidenced by the retention of intact open reading frames and functional signatures in genes encoding core alloherpesvirus proteins. Despite the intermittently faster evolution rate arising from active replication of the exogenous virus, the current host biogeographical constraints combined with the strong host specificity of herpesviruses (in the absence of rare host-jump events and development of alternative host reservoirs) would maintain the observed intra-species genetic homogeneity and the deep evolutionary relationship to the ancestral integrant observed in AciHV-3 sequences from different sturgeon species.

The biological consequences for sturgeon carriers of the ciAciHV-3 genome remain unknown. Future studies to determine if the viral genome has the capacity to reactivate to an actively replicating exogenous virus will be a critical first step towards understanding the potential impact of the ciAciHV-3 carrier status on sturgeon species. The work described herein and future research to address the many knowledge gaps pertaining to the ciAciHV-3 genome may be informative to decision making related to sturgeon stocks managed for conservation or commercial purposes. Specifically, the ubiquitous presence of the same genotype of AciHV-3 in sturgeon populations in HBDB eliminates the risk of introducing the virus genome into naïve stocks as all adult Lake Sturgeon appear to be carriers. This feature is relevant when stocks from one river are used to supplement stocks from another river within the basin. The same rationale can be applied to inter-basin transfers of Lake Sturgeon between the Hudson Bay and Atlantic basins, but additional testing with larger numbers of Lake Sturgeon samples will be required to verify the prevalence of AciHV-3 DNA in wild stocks from the Great Lakes and St. Lawrence River. A different strategy will be required for the inter-basin transfer of non-endemic sturgeon species given the observed genetic differences in AciHV-3 lineages from different hosts and the hallmark features of iciHVs evident from our limited analysis of AciHV-3 DNA in other sturgeon species. For example, the Q1mcp qPCR analysis revealed (1) the presence of a homologous AciHV-3 sequence in naïve spleen and skin sturgeon cell lines, both of which are derived from White Sturgeon tissues; (2) the high titer of AciHV-3 DNA (10^6.5^ epcs/ug DNA) detected in Green Sturgeon mucus is similar to the high titer of Lake Sturgeon AciHV-3 DNA found in other tissues (Data S9); and (3) a sequence homologous to the ciAciHV-3 LSGO genome is present in chromosome 48 of the Sterlet Sturgeon genome. This information suggests that endogenous AciHV-3 germline lineages are present in other sturgeon species. Thus, movement of species between drainage basins may result in vertical transmission of the ciAciHV-3 genome as a heritable genetic trait from parental DNA with unknown consequences. These considerations would also apply to sturgeon culture facilities housing multiple species of sturgeon on the same site but would likely only be relevant if cross-species hybrids were being produced. Studies evaluating the potential risk and consequences of a ciAciHV-3 genome inter-species host jump may provide useful information.

## Figures and Tables

**Table 1:** Polymorphisms in DNA sequences encoding 12 core alloherpesvirus proteins from contigs ptg001881l and ptg009622l relative to the acipenserid herpesvirus 3 LSGO genome.

Core Protein	Length of ORF (bp)		Number of Variations		
	Minimum	Maximum	Synonymous	Nonsynonymous	
				Conservative	Non-Conservative
Allo37	2064	2064	3	1	1
Allo54	1983	1983	1	0	1
Allo56	4383	4383	2	3	2
Allo60	1041	1041	4	0	1
Allo64	1710	1710	2	0	0
Capsid maturation protease	1782	1782	3	2	0
Capsid triplex protein 2	987	987	2	1	0
DNA polymerase catalytic subunit	4863	4863	1	0	1
Helicase primase helicase	1722	1722	5	1	0
Helicase primase primase	2202	2202	3	0	1
Major capsid protein	3885	3885	4	2	0
Terminase ATPase subunit	2274	2274	1	0	0
Total			31	10	7

**Table 2:** Acipenserid herpesvirus 3 (AciHV-3) qPCR testing of wild Lake Sturgeon *Acipenser fulvescens* broodstock, their gametes and larval progeny.

Brood Source	Sex	Year	AciHV-3 DNA Prevalence		
			(Number of Positive Sturgeon/Total Number of Sturgeon Tested (%))		
			Brood Fin	Gametes	Larvae (46–74 dpf)
Winnipeg River	Male	2011	2/2 (100)	2/2 (100)	-
	Female		3/3 (100)	3/3 (100)	
Landing River	Male	2012	2/2 (100)	2/2 (100)	-
	Female		2/2 (100)	2/2 (100)	
Winnipeg River	Male	2012	4/4 (100)	4/4 (100)	34/36 (94)
	Female		8/8 (100)	8/8 (100)	
Burntwood River	Male	2013	1/1 (100)	1/1 (100)	-
	Female		2/2 (100)	2/2 (100)	
Winnipeg River	Male	2013	4/4 (100)	4/4 (100)	24/24 (100)
	Female		5/5 (100)	5/5 (100)	
Landing River	Male	2014	4/4 (100)	4/4 (100)	-
	Female		2/2 (100)	1/1 (100)	

## Data Availability

All data are available in the manuscript and Supplementary Materials. DNA sequence data have been submitted to NCBI Genbank and assigned accession numbers as outlined in Table S2.

## Supplementary Materials

The following supporting information can be downloaded at: https://www.mdpi.com/article/10.3390/v17040534/s1, Figure S1: Q1mcp qPCR assay targets within artificial positive control (APC) plasmid pLSAciHV-3-AP; Figure S2: Acipenserid herpesvirus 3 genome features at the terminal ends of the unique (U) region; Figure S3: An overview of the internal repeat regions (IRRs) of the acipenserid herpesvirus 3 (AciHV-3) genome; Figure S4: Insertion site of the acipenserid herpesvirus 3 (AciHV-3) LSGO genome within the *Acipenser fulvescens* 28S–18S rRNA intergenic spacer region; Figure S5: Amino acid sequence alignments of full length sequence from the (A) capsid triplex subunit 2 protein, (B) capsid maturation protease and (C) terminase ATPase subunit 1; Figure S6: Q1mcp qPCR assay efficiency; Table S1: Prevalence of acipenserid herpesvirus 3 (AciHV-3) in wild Lake Sturgeon *Acipenser fulvescens* of the Hudson Bay drainage basin; Table S2: Virus taxa and other targets used to investigate acipenserid herpesvirus 3 LSGO systematics and Q1mcp qPCR performance; Table S3: Assembled contigs containing acipenserid herpesvirus 3 sequence; Table S4: Location and sequence of pac1-like or pac2-like sequences within the acipenserid herpesvirus 3 (AciHV-3) LSGO genome; Table S5: Annotation of acipenserid herpesvirus 3 (AciHV-3) LSGO genome; Table S6: Analytical repeatability of the Q1mcp qPCR assay; Data S1: Metadata for Allo-Malaco-Orthoherpesviruses used in orthology analyses; Data S2: DNA sequence data for 16 contigs evaluated in this study; Data S3: BLASTP and BLASTN analyses of host sequence; Data S4: Repeat data for each contig; Data S5: Amino acid sequence data for evaluating functional signatures of core proteins involved in virion morphogenesis; Data S6: DNA sequence data used for variant analyses; Data S7: Amino acid sequence data for reconstructing the Bayesian phylogeny of acipenserid herpesvirus 3; Data S8: Q1mcp qPCR data from the analytical validation study; Data S9: Acipenserid herpesvirus 3 (AciHV-3) DNA titer and mapping coordinates; Data S10: DNA sequence data for acipenserid herpesvirus 3 (AciHV-3) genotyping analyses.
