# Supplementary material for: Genomic and Epidemiological Investigations Reveal Chromosomal Integration of the Acipenserid Herpesvirus 3 Genome in Lake Sturgeon Acipenser fulvescens
Source: Viruses. 2025 Apr 5;17(4):534. doi: 10.3390/v17040534 (PMC12031113; doi:10.3390/v17040534)
Supplement: Supplementary file 1 [file viruses-17-00534-s001.zip › S1 Fig rev rnd2 prf.pptx]

## Slide 1
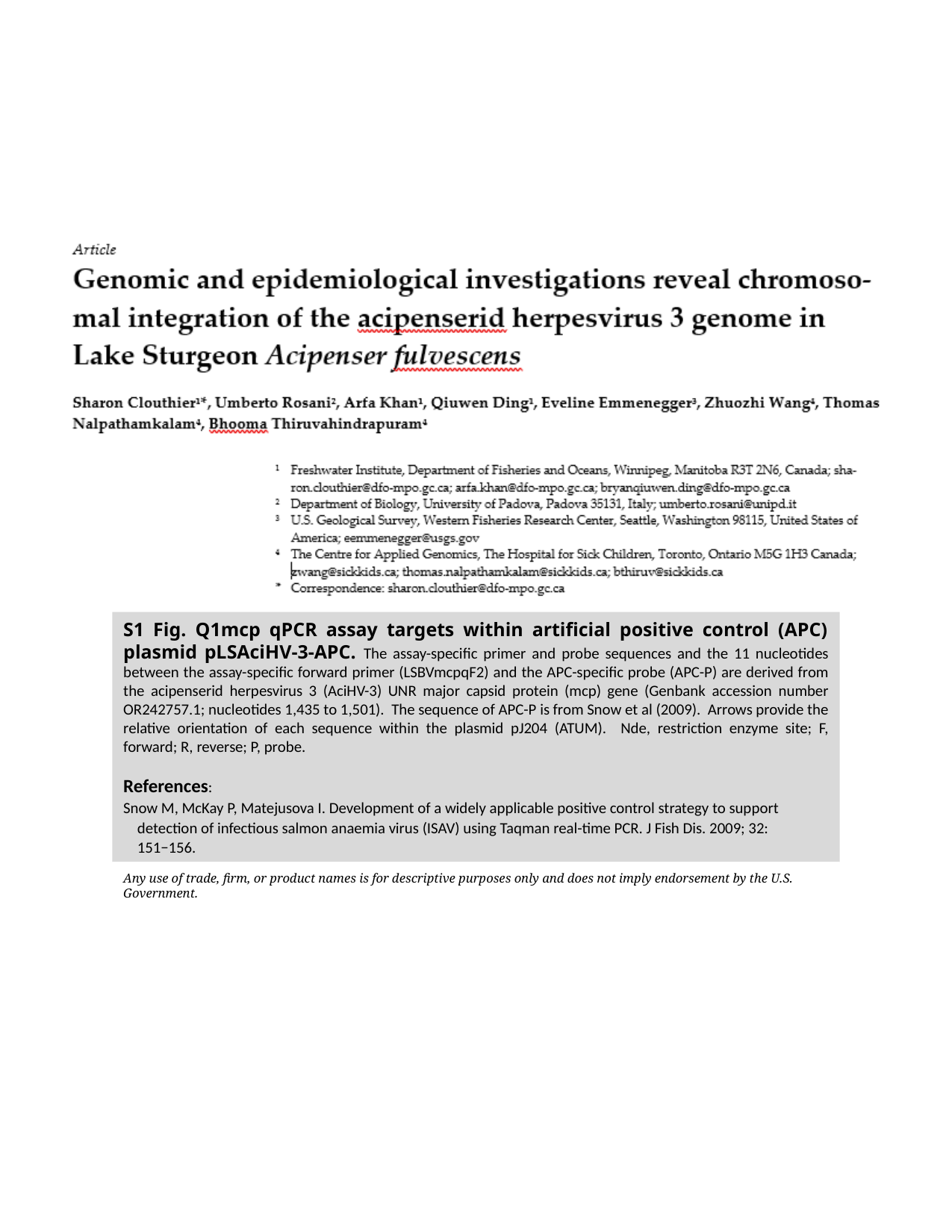

S1 Fig. Q1mcp qPCR assay targets within artificial positive control (APC) plasmid pLSAciHV-3-APC. The assay-specific primer and probe sequences and the 11 nucleotides between the assay-specific forward primer (LSBVmcpqF2) and the APC-specific probe (APC-P) are derived from the acipenserid herpesvirus 3 (AciHV-3) UNR major capsid protein (mcp) gene (Genbank accession number OR242757.1; nucleotides 1,435 to 1,501). The sequence of APC-P is from Snow et al (2009). Arrows provide the relative orientation of each sequence within the plasmid pJ204 (ATUM). Nde, restriction enzyme site; F, forward; R, reverse; P, probe.
References:
Snow M, McKay P, Matejusova I. Development of a widely applicable positive control strategy to support detection of infectious salmon anaemia virus (ISAV) using Taqman real-time PCR. J Fish Dis. 2009; 32: 151−156.
Any use of trade, firm, or product names is for descriptive purposes only and does not imply endorsement by the U.S. Government.

## Slide 2
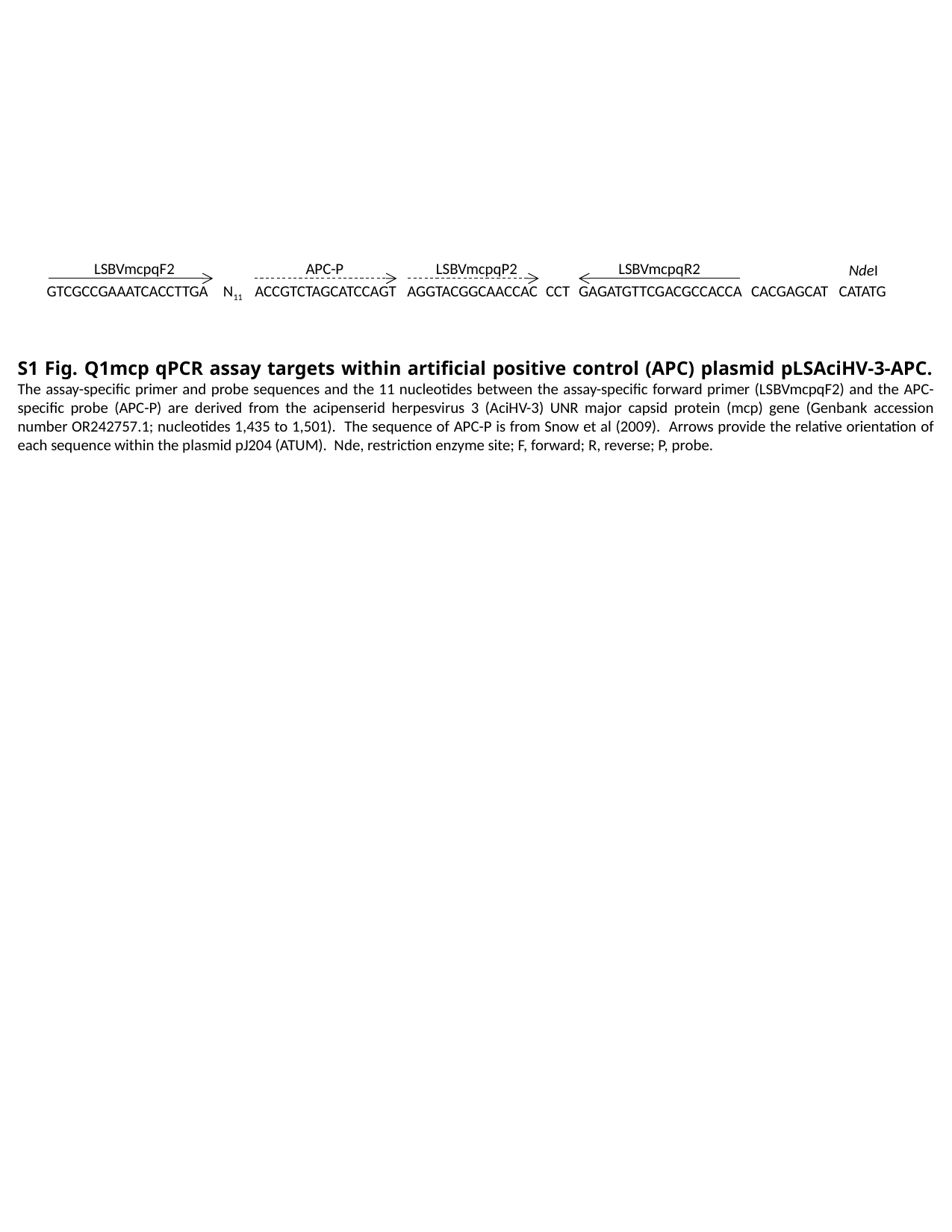

LSBVmcpqF2
APC-P
LSBVmcpqP2
LSBVmcpqR2
NdeI
GTCGCCGAAATCACCTTGA
N11
accgtctagcatccagt
AGGTACGGCAACCAC
Cct
GAGATGTTCGACGCCACCA
CACGAGCAT
CATATG
S1 Fig. Q1mcp qPCR assay targets within artificial positive control (APC) plasmid pLSAciHV-3-APC. The assay-specific primer and probe sequences and the 11 nucleotides between the assay-specific forward primer (LSBVmcpqF2) and the APC-specific probe (APC-P) are derived from the acipenserid herpesvirus 3 (AciHV-3) UNR major capsid protein (mcp) gene (Genbank accession number OR242757.1; nucleotides 1,435 to 1,501). The sequence of APC-P is from Snow et al (2009). Arrows provide the relative orientation of each sequence within the plasmid pJ204 (ATUM). Nde, restriction enzyme site; F, forward; R, reverse; P, probe.
