# Supplementary material for: Genomic and Epidemiological Investigations Reveal Chromosomal Integration of the Acipenserid Herpesvirus 3 Genome in Lake Sturgeon Acipenser fulvescens
Source: Viruses. 2025 Apr 5;17(4):534. doi: 10.3390/v17040534 (PMC12031113; doi:10.3390/v17040534)
Supplement: Supplementary file 1 [file viruses-17-00534-s001.zip › S2 Fig rev rnd2 prf.pptx]

## Slide 1
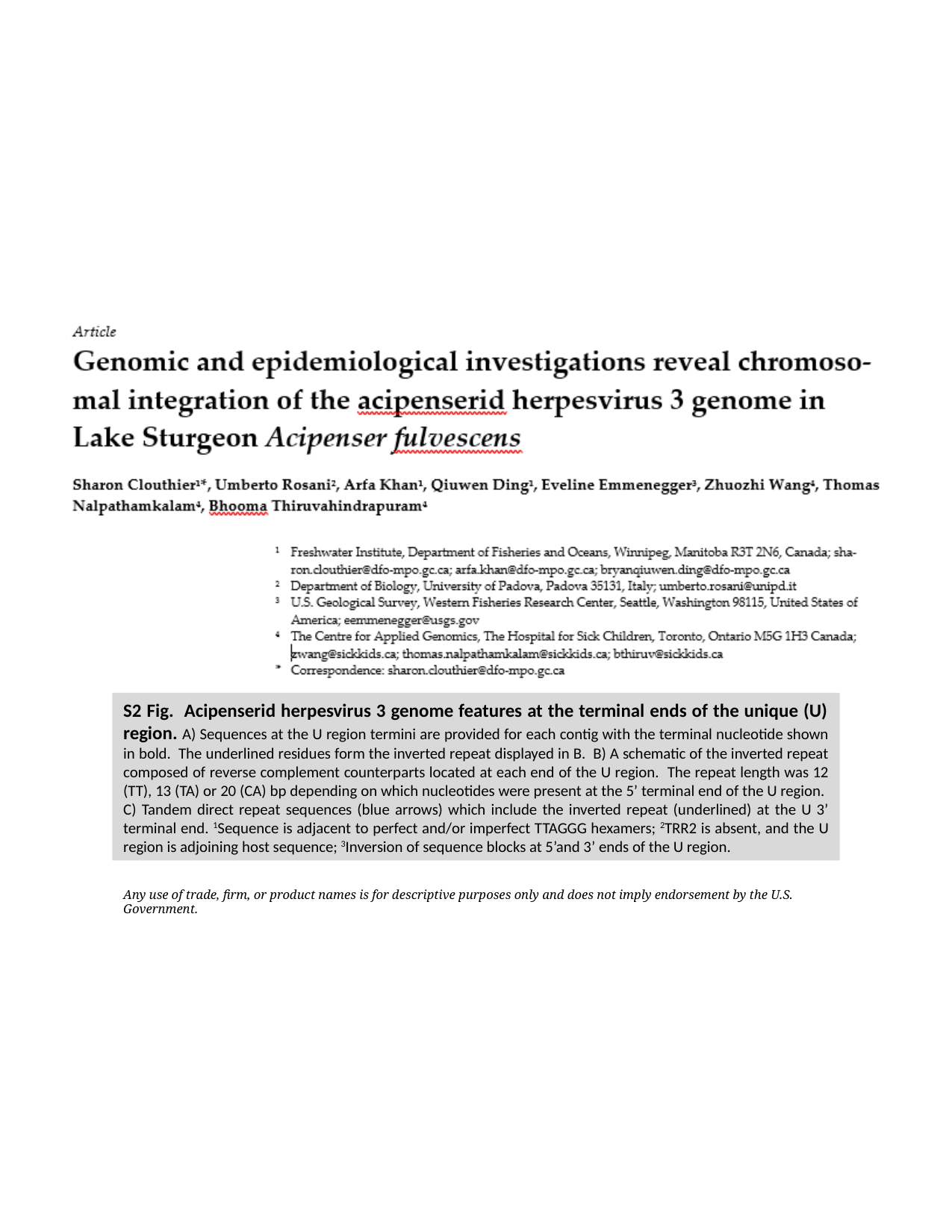

S2 Fig. Acipenserid herpesvirus 3 genome features at the terminal ends of the unique (U) region. A) Sequences at the U region termini are provided for each contig with the terminal nucleotide shown in bold. The underlined residues form the inverted repeat displayed in B. B) A schematic of the inverted repeat composed of reverse complement counterparts located at each end of the U region. The repeat length was 12 (TT), 13 (TA) or 20 (CA) bp depending on which nucleotides were present at the 5’ terminal end of the U region. C) Tandem direct repeat sequences (blue arrows) which include the inverted repeat (underlined) at the U 3’ terminal end. 1Sequence is adjacent to perfect and/or imperfect TTAGGG hexamers; 2TRR2 is absent, and the U region is adjoining host sequence; 3Inversion of sequence blocks at 5’and 3’ ends of the U region.
Any use of trade, firm, or product names is for descriptive purposes only and does not imply endorsement by the U.S. Government.

## Slide 2
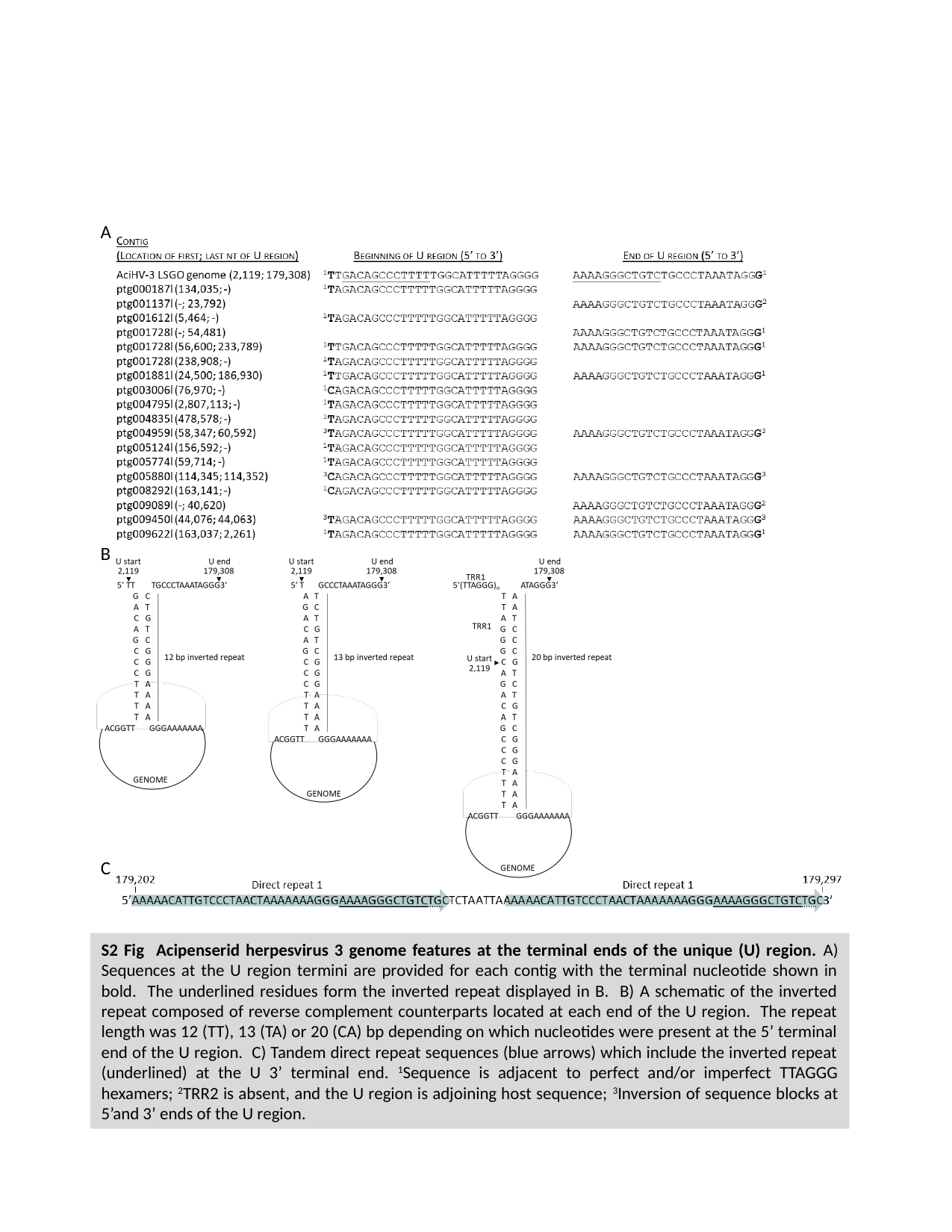

A
B
C
S2 Fig Acipenserid herpesvirus 3 genome features at the terminal ends of the unique (U) region. A) Sequences at the U region termini are provided for each contig with the terminal nucleotide shown in bold. The underlined residues form the inverted repeat displayed in B. B) A schematic of the inverted repeat composed of reverse complement counterparts located at each end of the U region. The repeat length was 12 (TT), 13 (TA) or 20 (CA) bp depending on which nucleotides were present at the 5’ terminal end of the U region. C) Tandem direct repeat sequences (blue arrows) which include the inverted repeat (underlined) at the U 3’ terminal end. 1Sequence is adjacent to perfect and/or imperfect TTAGGG hexamers; 2TRR2 is absent, and the U region is adjoining host sequence; 3Inversion of sequence blocks at 5’and 3’ ends of the U region.
