# Supplementary material for: Genomic and Epidemiological Investigations Reveal Chromosomal Integration of the Acipenserid Herpesvirus 3 Genome in Lake Sturgeon Acipenser fulvescens
Source: Viruses. 2025 Apr 5;17(4):534. doi: 10.3390/v17040534 (PMC12031113; doi:10.3390/v17040534)
Supplement: Supplementary file 1 [file viruses-17-00534-s001.zip › S3 Fig rev rnd2 prf.pptx]

## Slide 1
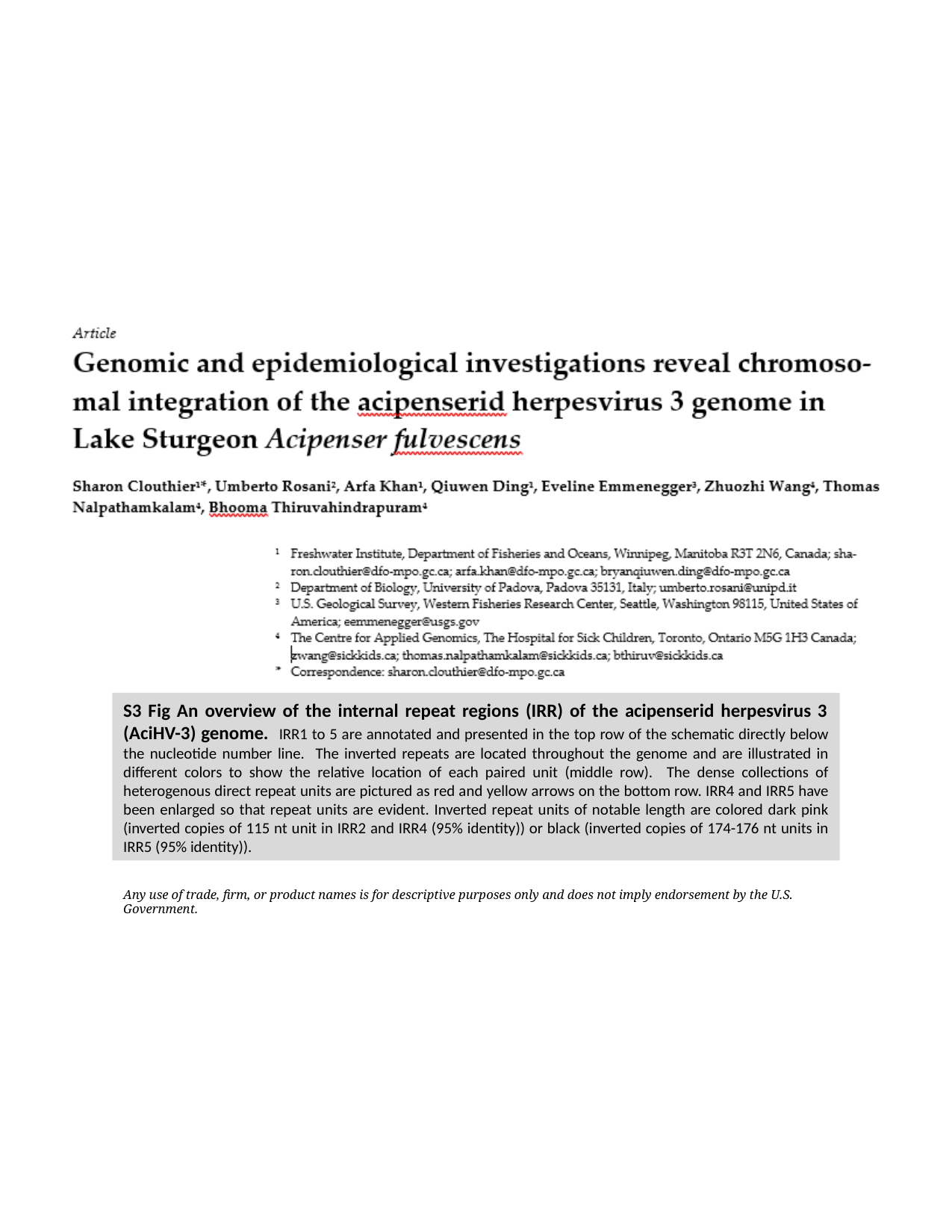

S3 Fig An overview of the internal repeat regions (IRR) of the acipenserid herpesvirus 3 (AciHV-3) genome. IRR1 to 5 are annotated and presented in the top row of the schematic directly below the nucleotide number line. The inverted repeats are located throughout the genome and are illustrated in different colors to show the relative location of each paired unit (middle row). The dense collections of heterogenous direct repeat units are pictured as red and yellow arrows on the bottom row. IRR4 and IRR5 have been enlarged so that repeat units are evident. Inverted repeat units of notable length are colored dark pink (inverted copies of 115 nt unit in IRR2 and IRR4 (95% identity)) or black (inverted copies of 174-176 nt units in IRR5 (95% identity)).
Any use of trade, firm, or product names is for descriptive purposes only and does not imply endorsement by the U.S. Government.

## Slide 2
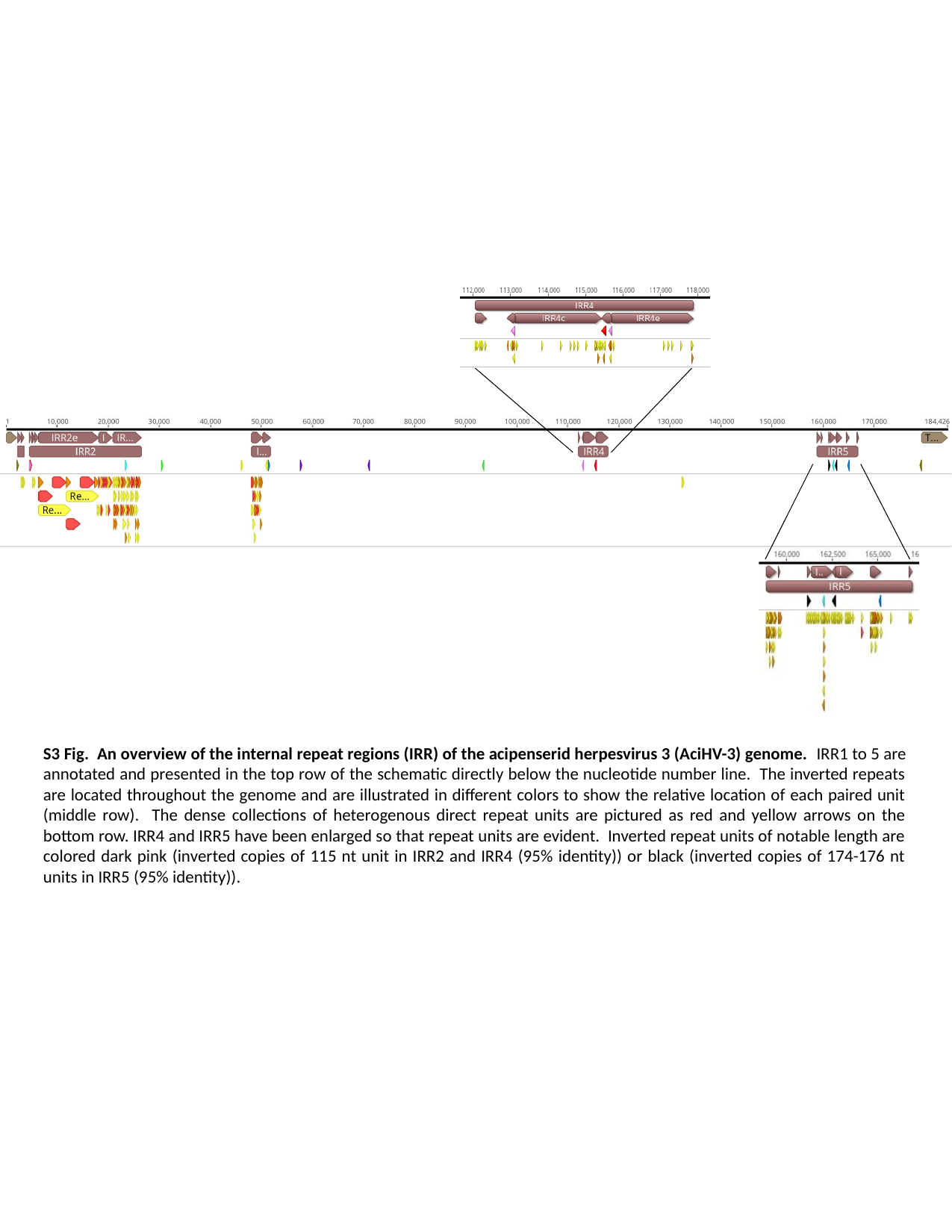

S3 Fig. An overview of the internal repeat regions (IRR) of the acipenserid herpesvirus 3 (AciHV-3) genome. IRR1 to 5 are annotated and presented in the top row of the schematic directly below the nucleotide number line. The inverted repeats are located throughout the genome and are illustrated in different colors to show the relative location of each paired unit (middle row). The dense collections of heterogenous direct repeat units are pictured as red and yellow arrows on the bottom row. IRR4 and IRR5 have been enlarged so that repeat units are evident. Inverted repeat units of notable length are colored dark pink (inverted copies of 115 nt unit in IRR2 and IRR4 (95% identity)) or black (inverted copies of 174-176 nt units in IRR5 (95% identity)).
