# Supplementary material for: Genomic and Epidemiological Investigations Reveal Chromosomal Integration of the Acipenserid Herpesvirus 3 Genome in Lake Sturgeon Acipenser fulvescens
Source: Viruses. 2025 Apr 5;17(4):534. doi: 10.3390/v17040534 (PMC12031113; doi:10.3390/v17040534)
Supplement: Supplementary file 1 [file viruses-17-00534-s001.zip › S4 Fig rev rnd2 prf.pptx]

## Slide 1
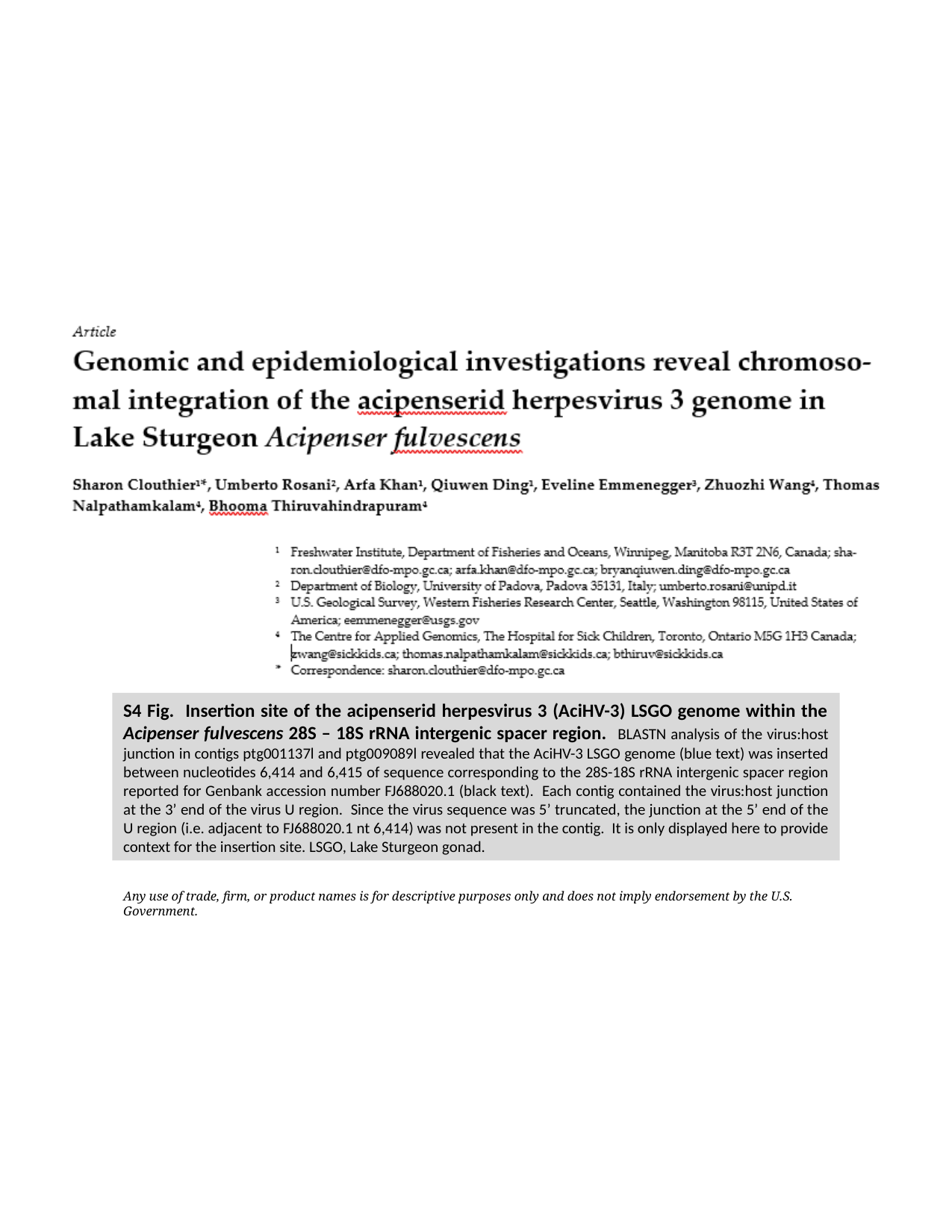

S4 Fig. Insertion site of the acipenserid herpesvirus 3 (AciHV-3) LSGO genome within the Acipenser fulvescens 28S – 18S rRNA intergenic spacer region. BLASTN analysis of the virus:host junction in contigs ptg001137l and ptg009089l revealed that the AciHV-3 LSGO genome (blue text) was inserted between nucleotides 6,414 and 6,415 of sequence corresponding to the 28S-18S rRNA intergenic spacer region reported for Genbank accession number FJ688020.1 (black text). Each contig contained the virus:host junction at the 3’ end of the virus U region. Since the virus sequence was 5’ truncated, the junction at the 5’ end of the U region (i.e. adjacent to FJ688020.1 nt 6,414) was not present in the contig. It is only displayed here to provide context for the insertion site. LSGO, Lake Sturgeon gonad.
Any use of trade, firm, or product names is for descriptive purposes only and does not imply endorsement by the U.S. Government.

## Slide 2
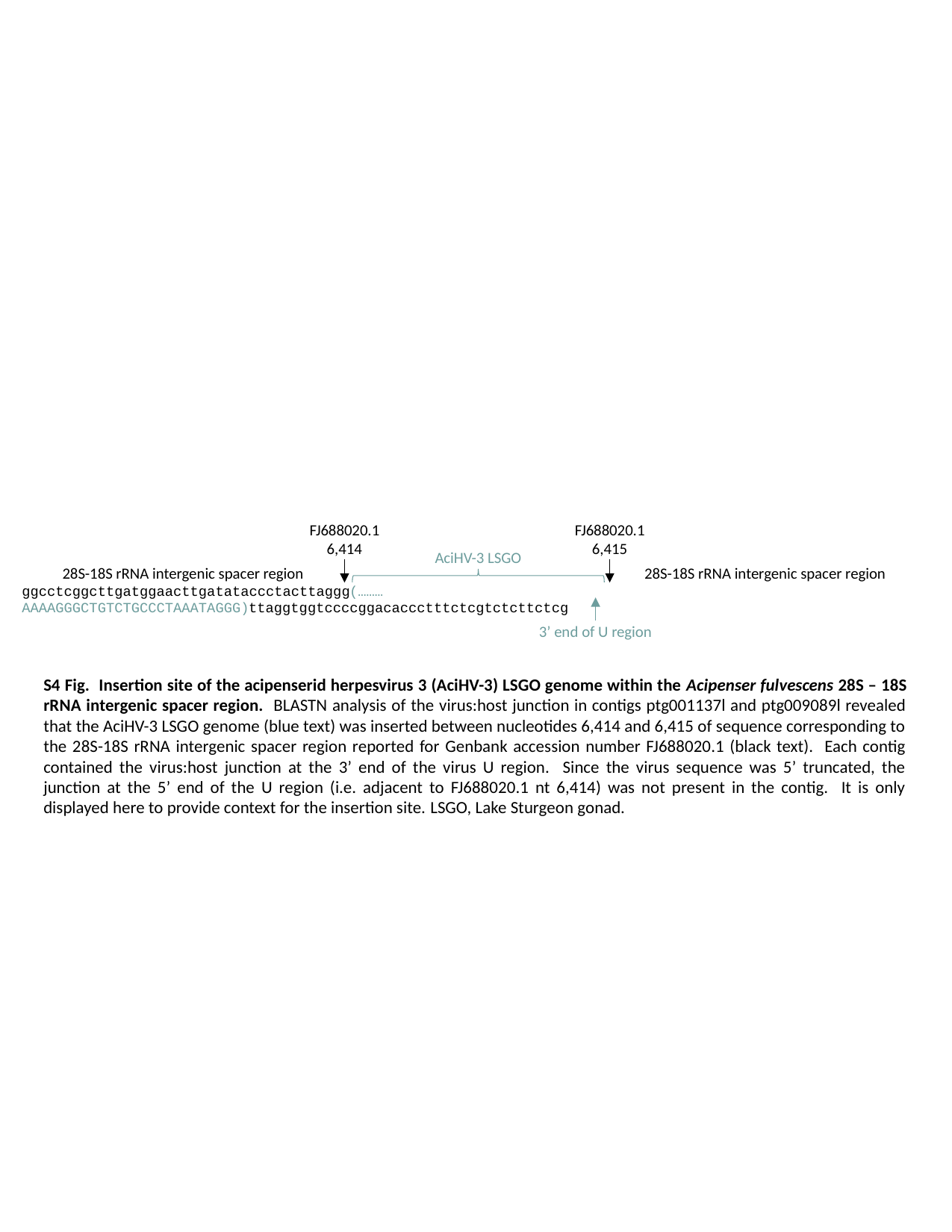

FJ688020.1
6,414
FJ688020.1
6,415
AciHV-3 LSGO
28S-18S rRNA intergenic spacer region
28S-18S rRNA intergenic spacer region
ggcctcggcttgatggaacttgatataccctacttaggg(………AAAAGGGCTGTCTGCCCTAAATAGGG)ttaggtggtccccggacaccctttctcgtctcttctcg
3’ end of U region
S4 Fig. Insertion site of the acipenserid herpesvirus 3 (AciHV-3) LSGO genome within the Acipenser fulvescens 28S – 18S rRNA intergenic spacer region. BLASTN analysis of the virus:host junction in contigs ptg001137l and ptg009089l revealed that the AciHV-3 LSGO genome (blue text) was inserted between nucleotides 6,414 and 6,415 of sequence corresponding to the 28S-18S rRNA intergenic spacer region reported for Genbank accession number FJ688020.1 (black text). Each contig contained the virus:host junction at the 3’ end of the virus U region. Since the virus sequence was 5’ truncated, the junction at the 5’ end of the U region (i.e. adjacent to FJ688020.1 nt 6,414) was not present in the contig. It is only displayed here to provide context for the insertion site. LSGO, Lake Sturgeon gonad.
