# Supplementary material for: Genomic and Epidemiological Investigations Reveal Chromosomal Integration of the Acipenserid Herpesvirus 3 Genome in Lake Sturgeon Acipenser fulvescens
Source: Viruses. 2025 Apr 5;17(4):534. doi: 10.3390/v17040534 (PMC12031113; doi:10.3390/v17040534)
Supplement: Supplementary file 1 [file viruses-17-00534-s001.zip › S5 Fig rev rnd2 prf.pptx]

## Slide 1
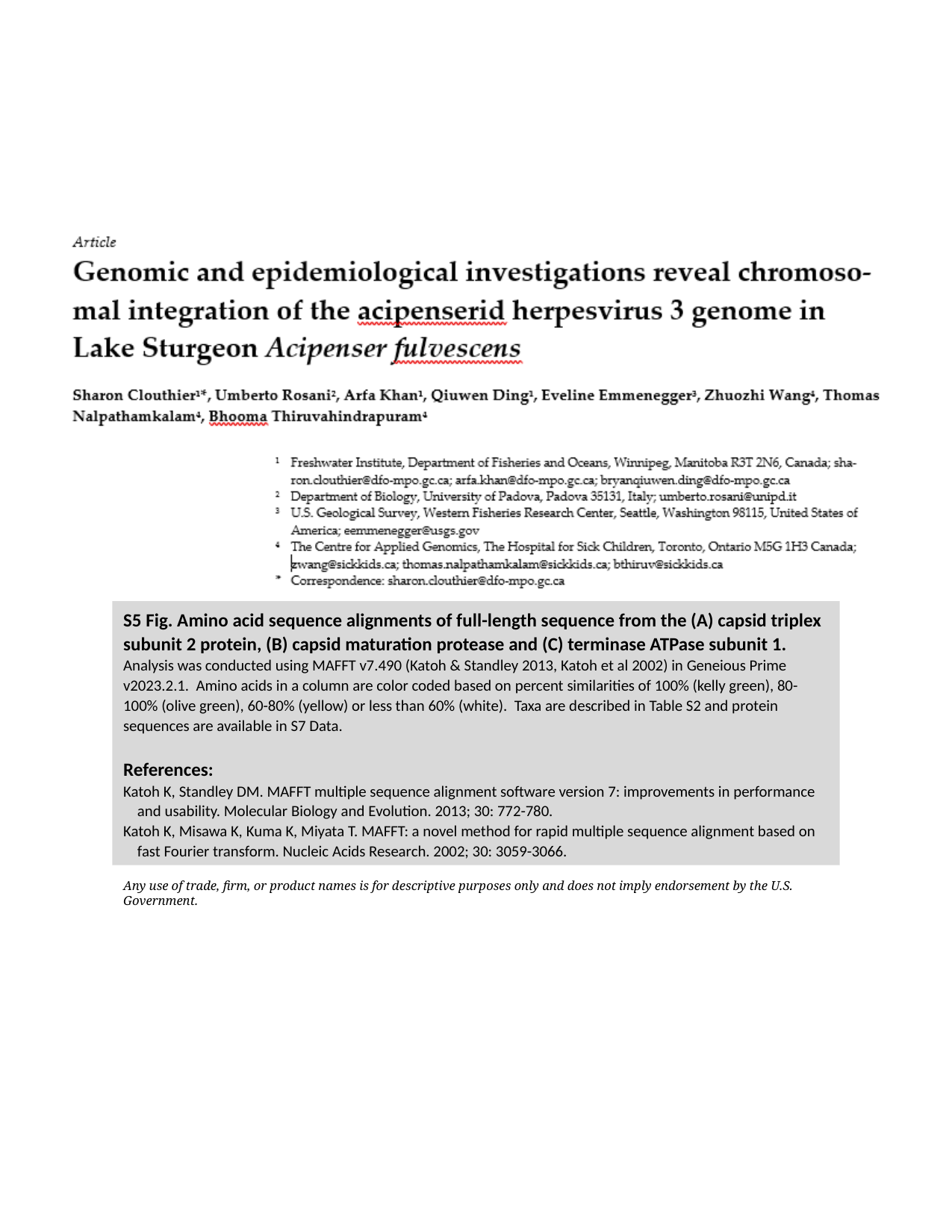

S5 Fig. Amino acid sequence alignments of full-length sequence from the (A) capsid triplex subunit 2 protein, (B) capsid maturation protease and (C) terminase ATPase subunit 1. Analysis was conducted using MAFFT v7.490 (Katoh & Standley 2013, Katoh et al 2002) in Geneious Prime v2023.2.1. Amino acids in a column are color coded based on percent similarities of 100% (kelly green), 80-100% (olive green), 60-80% (yellow) or less than 60% (white). Taxa are described in Table S2 and protein sequences are available in S7 Data.
References:
Katoh K, Standley DM. MAFFT multiple sequence alignment software version 7: improvements in performance and usability. Molecular Biology and Evolution. 2013; 30: 772-780.
Katoh K, Misawa K, Kuma K, Miyata T. MAFFT: a novel method for rapid multiple sequence alignment based on fast Fourier transform. Nucleic Acids Research. 2002; 30: 3059-3066.
Any use of trade, firm, or product names is for descriptive purposes only and does not imply endorsement by the U.S. Government.

## Slide 2
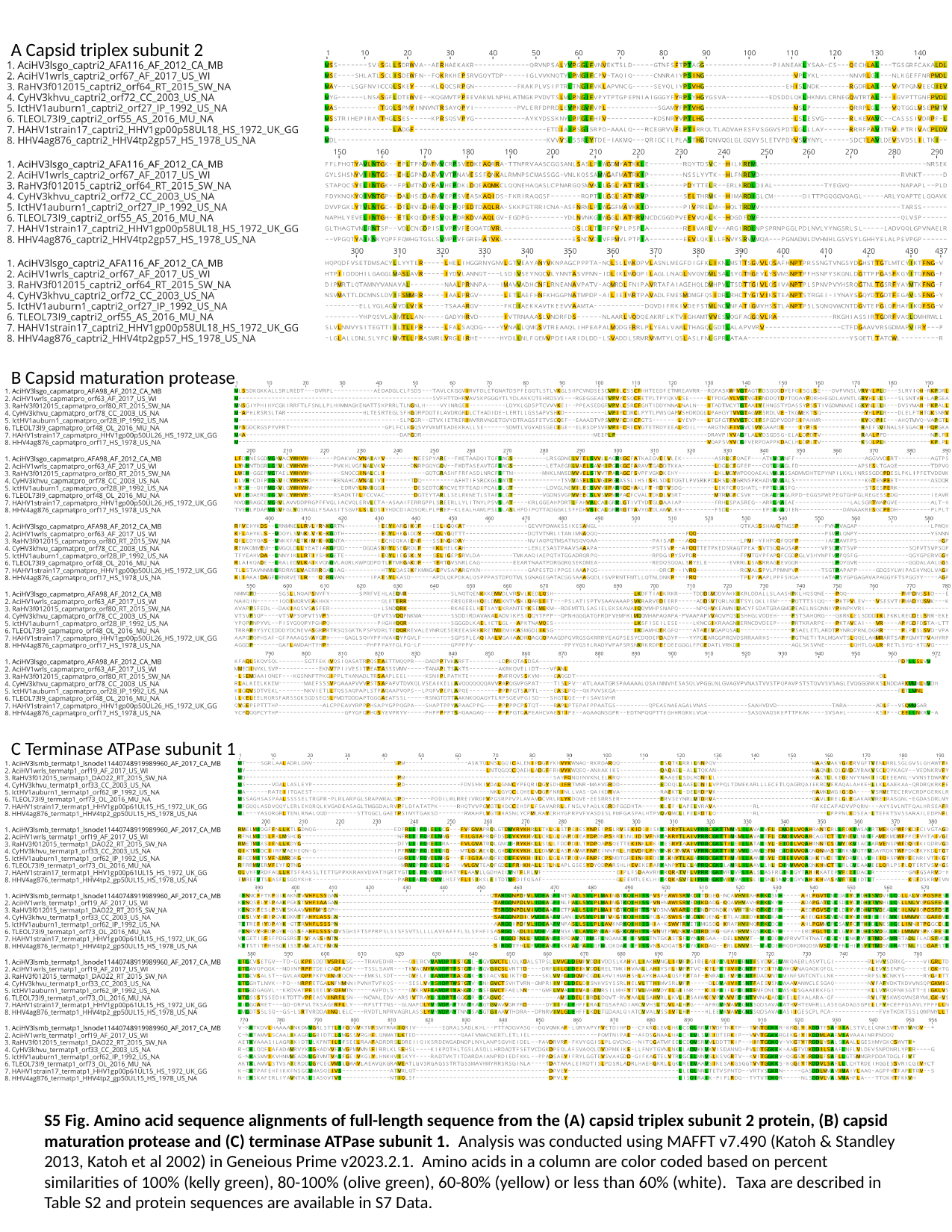

A Capsid triplex subunit 2
B Capsid maturation protease
C Terminase ATPase subunit 1
S5 Fig. Amino acid sequence alignments of full-length sequence from the (A) capsid triplex subunit 2 protein, (B) capsid maturation protease and (C) terminase ATPase subunit 1. Analysis was conducted using MAFFT v7.490 (Katoh & Standley 2013, Katoh et al 2002) in Geneious Prime v2023.2.1. Amino acids in a column are color coded based on percent similarities of 100% (kelly green), 80-100% (olive green), 60-80% (yellow) or less than 60% (white). Taxa are described in Table S2 and protein sequences are available in S7 Data.
