# Supplementary material for: Genomic and Epidemiological Investigations Reveal Chromosomal Integration of the Acipenserid Herpesvirus 3 Genome in Lake Sturgeon Acipenser fulvescens
Source: Viruses. 2025 Apr 5;17(4):534. doi: 10.3390/v17040534 (PMC12031113; doi:10.3390/v17040534)
Supplement: Supplementary file 1 [file viruses-17-00534-s001.zip › S6 Fig rev rnd2 prf.pptx]

## Slide 1
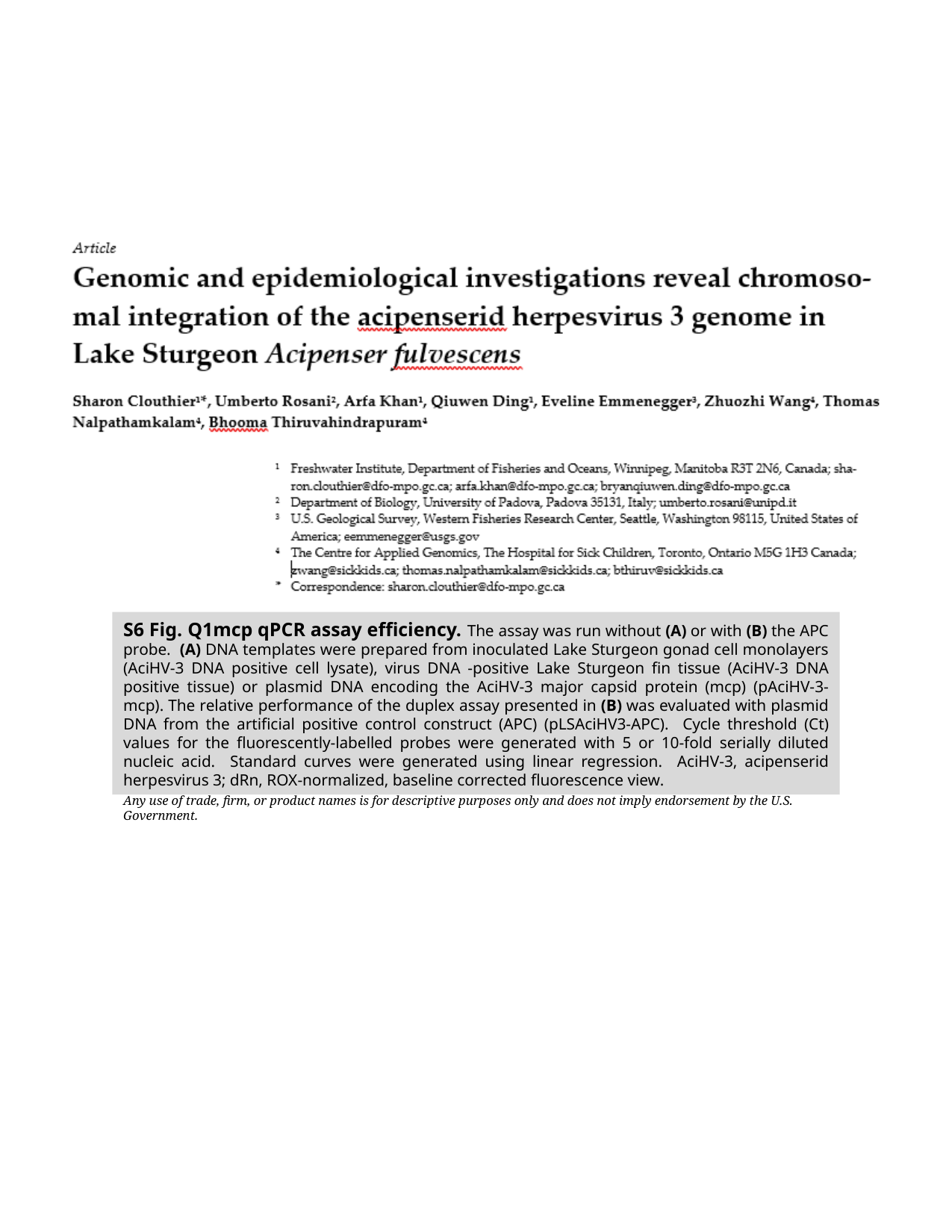

S6 Fig. Q1mcp qPCR assay efficiency. The assay was run without (A) or with (B) the APC probe. (A) DNA templates were prepared from inoculated Lake Sturgeon gonad cell monolayers (AciHV-3 DNA positive cell lysate), virus DNA -positive Lake Sturgeon fin tissue (AciHV-3 DNA positive tissue) or plasmid DNA encoding the AciHV-3 major capsid protein (mcp) (pAciHV-3-mcp). The relative performance of the duplex assay presented in (B) was evaluated with plasmid DNA from the artificial positive control construct (APC) (pLSAciHV3-APC). Cycle threshold (Ct) values for the fluorescently-labelled probes were generated with 5 or 10-fold serially diluted nucleic acid. Standard curves were generated using linear regression. AciHV-3, acipenserid herpesvirus 3; dRn, ROX-normalized, baseline corrected fluorescence view.
Any use of trade, firm, or product names is for descriptive purposes only and does not imply endorsement by the U.S. Government.

## Slide 2
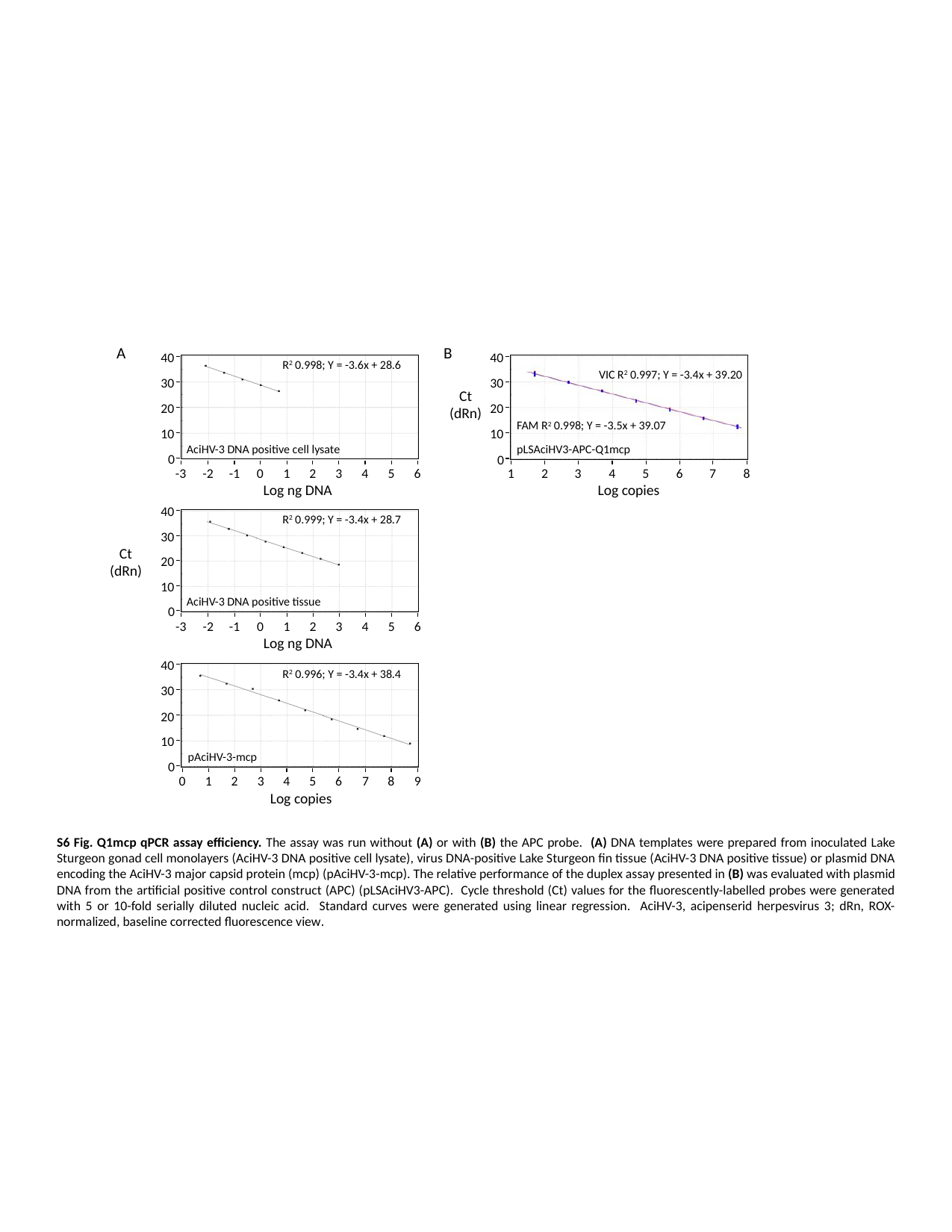

A
B
40
30
20
10
0
40
30
20
10
0
R2 0.998; Y = -3.6x + 28.6
VIC R2 0.997; Y = -3.4x + 39.20
Ct
(dRn)
FAM R2 0.998; Y = -3.5x + 39.07
AciHV-3 DNA positive cell lysate
pLSAciHV3-APC-Q1mcp
1
2
3
4
5
6
7
8
-3
-2
-1
0
1
2
3
4
5
6
Log ng DNA
Log copies
40
30
20
10
0
-3
-2
-1
0
1
2
3
4
5
6
R2 0.999; Y = -3.4x + 28.7
AciHV-3 DNA positive tissue
Log ng DNA
Ct
(dRn)
40
30
20
10
0
R2 0.996; Y = -3.4x + 38.4
pAciHV-3-mcp
0
1
2
3
4
5
6
7
8
9
Log copies
S6 Fig. Q1mcp qPCR assay efficiency. The assay was run without (A) or with (B) the APC probe. (A) DNA templates were prepared from inoculated Lake Sturgeon gonad cell monolayers (AciHV-3 DNA positive cell lysate), virus DNA-positive Lake Sturgeon fin tissue (AciHV-3 DNA positive tissue) or plasmid DNA encoding the AciHV-3 major capsid protein (mcp) (pAciHV-3-mcp). The relative performance of the duplex assay presented in (B) was evaluated with plasmid DNA from the artificial positive control construct (APC) (pLSAciHV3-APC). Cycle threshold (Ct) values for the fluorescently-labelled probes were generated with 5 or 10-fold serially diluted nucleic acid. Standard curves were generated using linear regression. AciHV-3, acipenserid herpesvirus 3; dRn, ROX-normalized, baseline corrected fluorescence view.
